# Does Acute Caffeine Alter the Temporal Countermovement Jump Response Following Reactive Strength Index-Individualized Drop Jumps in Chinese Han Basketball Players with Different Habitual Caffeine Exposure? A Randomized, Double-Blind, Placebo-Controlled Crossover Trial

**DOI:** 10.3390/nu18172842

**Published:** 2026-08-29

**Authors:** Jiaxuan Sun, Mingrui Wang, Lun Chen, Jinda Li, Yuting Zhou, Mingze Li, Ming Chen, Jingzhi Leng, Apudumalike Mieryazi, Jiahao Zhang, Yena Wang, Xiupeng Li, Bing Zhang, Li Li, Yimin Zhang, Jun Wang

**Affiliations:** 1 Department of Exercise Physiology, Beijing Sport University, Beijing 100084, China2316@bsu.edu.cn (L.L.); bsuwangjun@bsu.edu.cn (J.W.); 2 Laboratory of Sports Stress and Adaptation, General Administration of Sport of China, Beijing Sport University, Beijing 100084, China; 3 Top-Tier Performance Physical Fitness Training Center, Beijing 100020, China; 4 School of Strength and Conditioning, Beijing Sport University, Beijing 100084, China; 5 China Volleyball College, Beijing Sport University, Beijing 100084, China; 6 Sports Coaching College, Beijing Sport University, Beijing 100084, China; 7 School of Sport Science, Key Laboratory of Sports and Physical Fitness of the Ministry of Education, Beijing Sport University, Beijing 100084, Chinaymzhangno1@163.com (Y.Z.); 8 School of Physical Education and Health, Linyi University, Linyi 276000, China; 9 School of Physical Education, Shaanxi Normal University, Xi’an 710119, China; 10 Division of Sports Science and Physical Education, Tsinghua University, Beijing 100084, China; bzhang@tsinghua.edu.cn

**Keywords:** caffeine, basketball, post-activation performance enhancement, reactive strength index, drop jump, countermovement jump, habitual caffeine exposure, individualized sports nutrition

## Abstract

**Background/Objectives:** Whether caffeine provides additional benefit when superimposed on individualized plyometric conditioning remains unclear. We examined whether 3 mg·kg^−1^ caffeine altered the temporal countermovement jump (CMJ) response following reactive strength index (RSI)-derived individualized drop-jump (DJ) conditioning and explored moderation by habitual caffeine exposure. **Methods:** Thirty Chinese Han male basketball players (median age, 22.0 years [IQR, 21.0–27.75]) entered a randomized, double-blind, placebo-controlled crossover trial; 17 reported no caffeine exposure and 13 reported exposure during the preceding 4 weeks. After caffeine (CAF; 3 mg·kg^−1^) or placebo (PLA), a 60-min absorption period, and warm-up, participants performed a post-supplement, pre-conditioning CMJ followed by 3 × 5 DJs. CMJ was assessed at 15 s, 3, 6, 9, and 12 min. The primary outcome was the Condition × Time effect on CMJ. All 30 randomized participants contributed data; three did not complete Period 2 because readiness criteria were not met, but Period 1 data were retained (57 participant-period observations). **Results:** CMJ varied across time (*p* < 0.001), but Condition × Time was not significant (*p* = 0.305). CAF–PLA differences at 15 s, 3, 6, 9, and 12 min were 0.89 cm (95% CI, −0.11 to 1.88), 0.39 (−0.61 to 1.38), 0.21 (−0.79 to 1.21), 0.84 (−0.15 to 1.84), and 0.71 (−0.29 to 1.71), respectively (all Holm-adjusted *p* ≥ 0.399). Habitual caffeine exposure showed no global moderation continuously (*p* = 0.158) or categorically (*p* = 0.116). An isolated 9-min contrast at zero reported exposure favored CAF by 1.83 cm (95% CI, 0.74–2.92; Holm-adjusted *p* = 0.031) but was exploratory. RPE showed no Condition × Time interaction (*p* = 0.253); at 15 s, CAF–PLA was −0.60 points (95% CI, −1.09 to −0.12; Holm-adjusted *p* = 0.088). **Conclusions:** This study did not obtain clear evidence that 3 mg·kg^−1^ caffeine provided additional CMJ improvement following this specific RSI-derived individualized DJ protocol. Habitual caffeine exposure did not systematically moderate the temporal response; the isolated signal was exploratory.

## 1. Introduction

Basketball is a high-intensity intermittent team sport in which athletes repeatedly perform explosive actions such as jumping, acceleration, sprinting, deceleration, and rapid changes in direction. Accordingly, practical strategies capable of acutely optimizing neuromuscular performance before competition or during breaks may be valuable for basketball performance [1,2]. Post-activation performance enhancement (PAPE), generally referring to a transient improvement in subsequent voluntary performance following a conditioning activity (CA), has therefore received increasing attention in sports characterized by high explosive demands [3,4]. Its magnitude is influenced by multiple factors, including CA type and dose, athlete characteristics, and recovery duration [3,5].

Plyometric CAs are particularly relevant to basketball because they involve rapid stretch–shortening cycle actions resembling many sport-specific movements while requiring less external loading and equipment than traditional heavy resistance-based CAs [3,6]. Among these approaches, drop jumps (DJs) provide a standardized eccentric–concentric stimulus closely related to jumping performance. However, prescribing the same drop height to all athletes may impose different mechanical demands. Multi-height DJ testing has therefore been used to identify the height associated with an individual’s highest reactive strength index (RSI), with previous work demonstrating good reliability of peak RSI and its corresponding drop height [7,8]. Importantly, the height associated with peak RSI during an isolated assessment should not be assumed to represent the optimal conditioning height for subsequently eliciting PAPE during repeated DJs. Accordingly, the present study used peak RSI only as an operational criterion to derive an individualized DJ height, without assuming that this height provided an optimal potentiation–fatigue balance.

Caffeine is one of the most extensively investigated acute ergogenic aids in exercise science. Its actions include antagonism of adenosine receptors and modulation of central nervous system activity, with potential effects on alertness, perceived effort, motor drive, and neuromuscular performance [9,10]. Acute doses of approximately 3–6 mg·kg^−1^ have been associated with improvements in several strength-, power-, sprint-, and jump-related outcomes, although considerable inter-individual variability exists [9]. For example, 3 mg·kg^−1^ caffeine has been reported to improve CMJ height and several force–velocity-related variables during acute testing [11].

However, evidence that caffeine independently enhances performance does not establish that it will provide an additional benefit when superimposed on a conditioning activity. Direct studies combining caffeine supplementation with PAPE-oriented conditioning protocols remain limited and have produced inconsistent findings. Filip-Stachnik et al. found no clear additional CMJ benefit of caffeine when combined with a heavy-squat CA [12], whereas a recent study in Chinese male collegiate basketball players reported that 5 mg·kg^−1^ caffeine combined with a complex PAPE protocol improved Wingate performance relative to PAPE plus placebo [13]. Thus, a more specific question remains unresolved: when the same individually prescribed plyometric conditioning stimulus is applied under both caffeine and placebo conditions, does a moderate dose of caffeine further alter the subsequent temporal jump-performance response? Differences among previous studies in caffeine dose, conditioning characteristics, recovery timing, and performance task may contribute to the heterogeneous findings, but these possibilities remain hypotheses requiring direct investigation.

Habitual caffeine exposure may represent an additional source of inter-individual variability. Repeated caffeine exposure can induce adaptations within the adenosinergic system, although whether these changes meaningfully attenuate the acute ergogenic response during exercise remains uncertain [10,14]. Several studies have reported little evidence that habitual caffeine consumption substantially modifies acute exercise responses [15,16,17], whereas a recent systematic review and meta-analysis suggested that individuals with lower habitual exposure may experience larger improvements in some strength–power outcomes [18]. Habitual caffeine exposure should therefore be considered a plausible but unresolved moderator of acute caffeine responsiveness rather than an established determinant.

Caffeine exposure in China arises from multiple dietary sources, including coffee, traditional tea and tea-based beverages, energy drinks, and caffeinated soft drinks [19]. Restricting the present sample to Chinese Han athletes also provided a methodological means of defining a relatively homogeneous study population. China comprises populations with diverse ethnic and ancestral backgrounds, and large-scale genomic studies have demonstrated population structure and allele-frequency variation across Chinese populations and within Han Chinese [20,21]. Such heterogeneity may be relevant because genetic variation in caffeine metabolism and sensitivity pathways, including CYP1A2 and ADORA2A, has been investigated as a potential contributor to individual caffeine responses [22,23]. Thus, recruitment was restricted to self-reported Han Chinese athletes to reduce unmeasured ancestry-related heterogeneity, rather than because Han ethnicity was assumed to modify caffeine efficacy; ancestry and caffeine-related genotypes were not directly assessed in the present study.

Accordingly, the present randomized, double-blind, placebo-controlled crossover study investigated whether acute ingestion of 3 mg·kg^−1^ caffeine altered the temporal CMJ response following a 3 × 5 RSI-derived individualized DJ conditioning protocol in trained Chinese Han male basketball players. Because both experimental conditions received the same DJ conditioning activity and no no-conditioning condition was included, the study was designed to estimate the additional within-participant effect of caffeine relative to placebo within this specific conditioning context, rather than to determine the independent causal effect of the DJ conditioning itself. Furthermore, Pre-CA CMJ was assessed after supplement ingestion and the standardized warm-up and therefore represents a post-supplement, pre-conditioning reference rather than a true pre-supplementation baseline; consequently, the present design does not estimate the complete acute effect of caffeine from before ingestion.

The primary hypothesis was that caffeine would modify the post-conditioning temporal CMJ trajectory relative to placebo, statistically expressed as a Condition × Time interaction across the five prespecified post-conditioning time points. Habitual caffeine exposure was examined as an exploratory moderator, with the hypothesis that it might modify this Condition × Time response. HR and RPE were prespecified as secondary outcomes, whereas maximum observed ΔCMJ, 0–12 min ΔCMJ AUC, and time to maximum observed ΔCMJ were evaluated as exploratory derived outcomes. These secondary and exploratory analyses were considered non-confirmatory and interpreted hierarchically relative to the primary Condition × Time hypothesis.

## 2. Materials and Methods

### 2.1. Study Design

**Figure 1:**
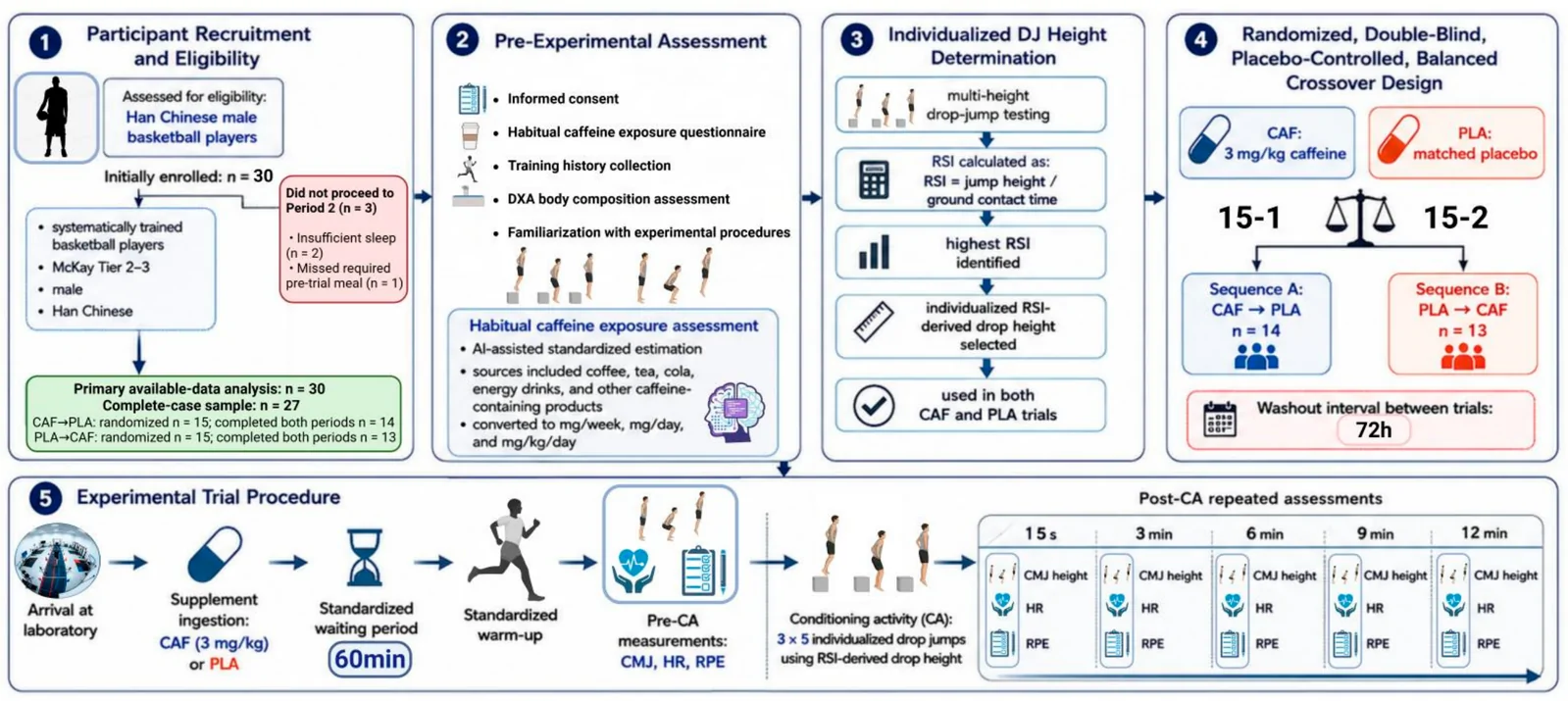
Study design and experimental workflow. CAF, caffeine; PLA, placebo; DJ, drop jump; RSI, reactive strength index; CA, conditioning activity; CMJ, countermovement jump; HR, heart rate; RPE, rating of perceived exertion; DXA, dual-energy X-ray absorptiometry.

This study employed a randomized, double-blind, placebo-controlled, counterbalanced crossover design to investigate the effects of acute caffeine ingestion on the temporal countermovement jump (CMJ) response following reactive strength index (RSI)-individualized drop jump (DJ) conditioning, and to explore whether habitual caffeine exposure moderated these acute responses. Because both experimental conditions incorporated the same individualized DJ conditioning activity, the crossover comparison was designed to estimate the additional within-participant effect of caffeine relative to placebo on CMJ performance following conditioning. Accordingly, the study was not designed to independently determine the causal effect of DJ conditioning itself relative to a no-conditioning condition. The complete study workflow is presented in Figure 1.

Before the formal crossover trials, participants completed baseline assessments, habitual caffeine exposure assessment, DXA-derived body composition assessment, familiarization with the experimental procedures, and multi-height DJ testing. The drop height that elicited the highest RSI for each participant was identified and subsequently used as the individualized DJ conditioning height in both experimental trials.

A total of 30 participants were randomly allocated in a 1:1 ratio to two intervention sequences within a two-treatment, two-period Latin square counterbalanced crossover design: caffeine followed by placebo (CAF→PLA) or placebo followed by caffeine (PLA→CAF), with 15 participants initially assigned to each sequence. Each participant was assigned a unique study identification number, after which intervention sequences were generated using the random-number function in Microsoft Excel, with allocation constrained to maintain equal numbers in the two sequences. The allocation schedule was prepared and retained by an independent researcher who was not involved in the experimental procedures. To maintain allocation concealment and double blinding, CAF and PLA were provided in identically presented capsules and administered according to pre-generated treatment codes. Participants and on-site testing personnel remained unaware of treatment allocation until completion of data collection and prespecified primary data processing.

The two experimental trials were separated by a 72-h washout period. Given the approximately 4–6-h average elimination half-life of caffeine in adults, despite substantial inter-individual variability, this interval was considered sufficient to minimize residual acute pharmacological effects of caffeine while also allowing adequate recovery from the preceding DJ conditioning activity and repeated maximal CMJ assessments [9]. To minimize potential circadian influences on neuromuscular performance, all experimental trials were conducted within a standardized afternoon testing window centered at approximately 15:00 (approximately 14:00–16:00), and each participant completed both crossover trials within this same time window. Potential period- and sequence-related influences inherent to the crossover design were accounted for in the statistical modelling.

During each experimental trial, participants ingested caffeine (CAF; 3 mg·kg^−1^ body mass) or a matched placebo (PLA). Following a standardized 60-min absorption period, participants completed an identical standardized warm-up, after which a pre-conditioning (Pre-CA) CMJ assessment was performed. Because the Pre-CA CMJ was obtained after supplement ingestion and the standardized warm-up, it was treated as a period-specific pre-conditioning reference value rather than a pre-supplementation baseline. Participants then completed the individualized DJ conditioning activity. CMJ performance was assessed at 15 s, 3 min, 6 min, 9 min, and 12 min after conditioning to characterize the post-conditioning temporal response, while heart rate (HR) and rating of perceived exertion (RPE) were recorded at the corresponding time points. All 30 randomized participants contributed data from at least one valid experimental period and were included in the primary available-data mixed-effects analysis; 27 participants completed both periods and comprised the complete-case sample (CAF→PLA, *n* = 14; PLA→CAF, *n* = 13).

### 2.2. Participants

Chinese Han male basketball players aged 18–35 years were recruited through the China Basketball College of Beijing Sport University Recruitment information was disseminated through posters, WeChat/WeChat Moments, and social-media platforms (e.g., Douyin). Potential participants underwent structured preliminary eligibility screening via WeChat and/or telephone before attending laboratory procedures. Written informed consent was obtained from all eligible participants before familiarization and experimental testing.

Eligible participants were required to have a systematic basketball-training background and competitive experience, including participation at the provincial or municipal level and/or experience within nationally recognized athlete grading systems. Training and performance caliber was additionally classified according to the framework proposed by McKay et al. [24], rather than relying solely on administrative athlete certification. McKay tier was used to characterize baseline training/performance caliber and was included as a categorical covariate in the longitudinal models. Current weekly training frequency and training duration were not prospectively collected as standardized study variables and were therefore not retrospectively reconstructed.

Preliminary screening covered general health, recent sport-related injury or pain, relevant medical history, medication and supplement use, caffeine intolerance or contraindications, and the ability to safely perform maximal jumping tasks. Participants were required to be free from known cardiovascular, respiratory, neuromuscular, or musculoskeletal disorders that could compromise participation in the standardized warm-up, drop-jump (DJ), or countermovement-jump (CMJ) procedures. Exclusion criteria included recent injury or pain affecting performance, use of medications or supplements with potential acute effects on performance, caffeine contraindication or marked intolerance, or any other condition considered to compromise participant safety or study validity. Eligibility and readiness for maximal-effort testing were reconfirmed before each experimental session.

To reduce acute behavioral variation between crossover trials, participants were instructed to obtain approximately 8 h of sleep on the night before testing, maintain their habitual dietary pattern, and consume their usual meals before each afternoon trial. Participants were required to abstain from caffeine-containing products and strenuous exercise for 24 h before testing and to avoid alcohol consumption, smoking, and other behaviors likely to acutely influence neuromuscular performance.

Compliance was assessed before each trial using a structured verbal readiness check addressing sleep, meal consumption, recent exercise, caffeine and alcohol intake, smoking, current injury or pain, and general physical readiness. Testing proceeded only when participants satisfied the pre-trial requirements and were considered suitable for maximal-effort testing. Compliance assessment was based on participant self-report and investigator verification rather than objective sleep monitoring, quantitative dietary assessment, or biochemical verification. The 24-h caffeine-abstinence requirement was applied uniformly across participants.

An a priori sample-size calculation was completed before recruitment for the primary Condition × Time hypothesis. The calculation was based on the expected temporal pattern of the within-participant CAF–PLA differences across the five prespecified post-conditioning time points, with α = 0.05 and a target statistical power of 80%. Under the prespecified planning assumptions described in Section 2.10, the minimum required sample size was 30 participants. Accordingly, the recruitment target was set at 30 participants, and 30 participants were subsequently recruited and randomized.

The study was approved by the Ethics Committee of Sport Science Experiments at Beijing Sport University (Ethics Approval No. 2026209H) and was conducted as a prespecified basketball-player substudy of a larger randomized crossover trial registered with the Chinese Clinical Trial Registry (ChiCTR2600124780; registered on 18 May 2026). All participants provided written informed consent after receiving an explanation of the study objectives, procedures, and potential risks.

### 2.3. DXA Body Composition Assessment

Before the formal crossover trials, all participants underwent a dual-energy X-ray absorptiometry (DXA) body composition assessment to obtain measures including body mass, body fat percentage, DXA-derived lean mass, and bone mineral density (BMD). The assessment was performed using a GE Lunar iDXA system (GE Healthcare, Madison, WI, USA). To minimize the potential influence of recent dietary intake, hydration status, and physical activity on body composition measurements, participants were instructed to avoid strenuous exercise before testing and completed the assessment under relatively standardized dietary and hydration conditions. During the assessment, participants wore lightweight, close-fitting clothing without metal components and removed jewelry, watches, belts, and other metallic objects that could interfere with scanning accuracy. Before each formal assessment, equipment quality control and calibration procedures were conducted according to the manufacturer’s recommendations, including routine quality assurance testing using a standard phantom. During scanning, participants were positioned in a supine position at the center of the scanning table, with the body naturally extended, arms positioned slightly away from the trunk, and lower limbs maintained in a standardized position. Whole-body measurements were performed according to the manufacturer’s standard scanning protocol. All scans were conducted by the same trained researcher following standardized operating procedures to minimize operator-related variability.

### 2.4. Assessment of Habitual Caffeine Exposure

**Figure 2:**
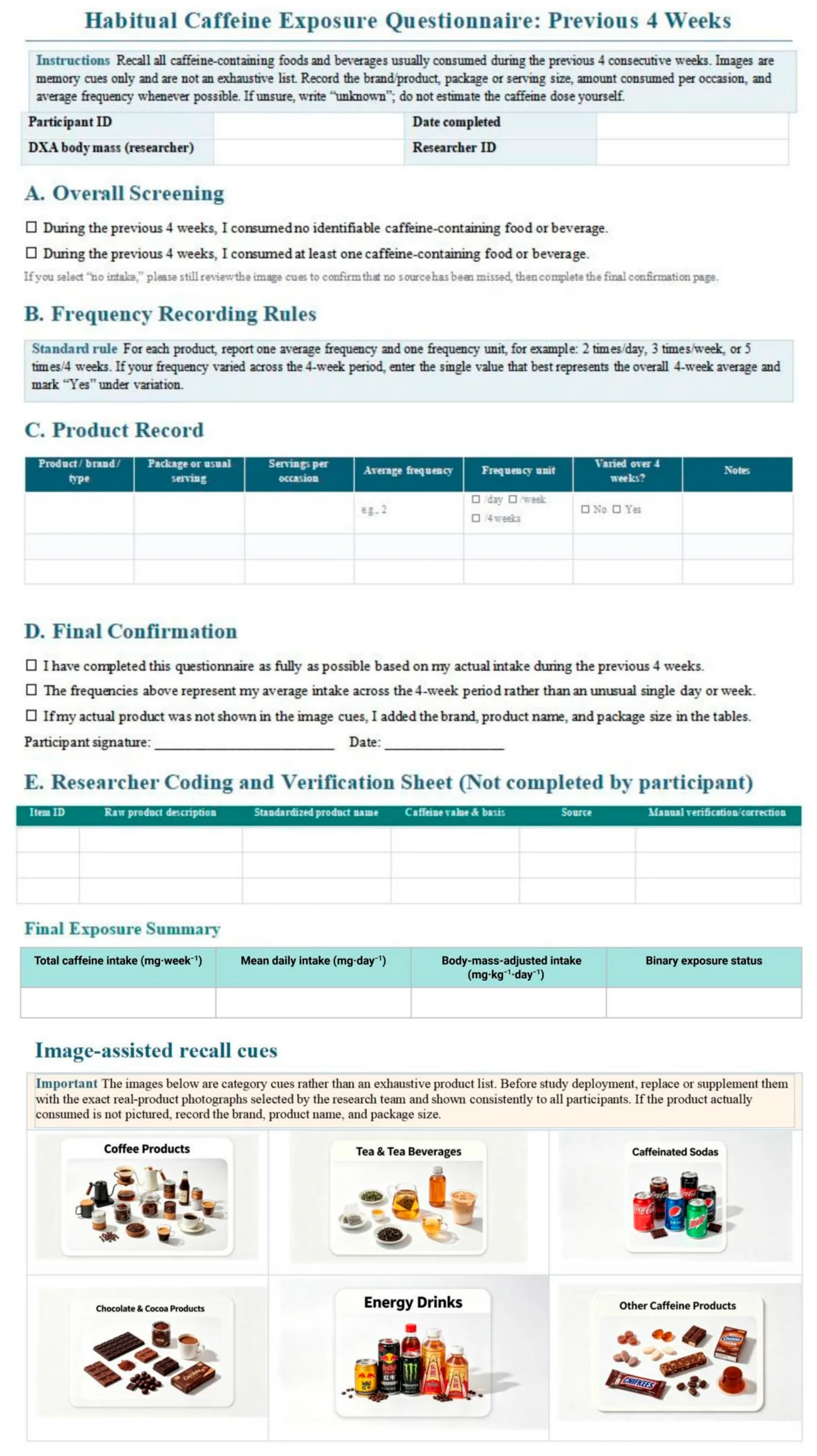
Habitual caffeine exposure questionnaire and image-assisted recall cues for the previous 4 weeks. The figure presents the English version of the questionnaire and image-assisted recall materials; an equivalent Chinese-language version was administered to participants during actual data collection.

Before the formal experiments, a structured Chinese questionnaire was used to assess participants’ caffeine intake during the preceding 4 consecutive weeks. The questionnaire was designed with reference to previously published caffeine-intake and food-frequency assessment methods [25,26,27] and covered common caffeine-containing products, including coffee, tea and tea-based beverages, energy drinks, cola beverages, and other common caffeine-containing foods or beverages. Participants reported the specific product or category, typical serving size, and average consumption frequency; when necessary, image-assisted recall was used to help identify products, packaging, and common serving sizes [28]. For products not covered by the image materials, additional information on brand, product name, intake amount, and consumption frequency was recorded. The questionnaire is presented in Figure 2. In addition, before formal use, the questionnaire underwent linguistic translation and cultural adaptation. The original questionnaire framework was translated into Chinese by a language professional and was subsequently independently reviewed by two bilingual experts with relevant professional backgrounds for semantic clarity, conceptual consistency, and cultural appropriateness. Before the formal study, the questionnaire was pretested with three individuals from the target population, and necessary wording was adjusted according to their feedback. This process followed cross-cultural adaptation principles involving professional translation, expert review, and target-population pretesting [29,30,31].

Caffeine exposure was primarily characterized as a continuous variable. Individual intake was estimated according to serving size, average consumption frequency over the preceding 4 weeks, and the corresponding caffeine content. Product-specific caffeine content was preferentially determined from product labels, nutritional information, or manufacturer information; when such information was unavailable, standardized reference values predefined by the research team were used. Intake was ultimately converted into mg·week^−1^, mg·day^−1^, and mg·kg^−1^·day^−1^. The primary exposure-moderation analysis used mg·kg^−1^·day^−1^, which was transformed using log(1 + x) and z-standardized before model fitting. The binary indicator was retained only as a secondary descriptive and sensitivity representation. Participants who reported no identifiable caffeine intake during the preceding 4 weeks were defined as having no reported caffeine exposure (0 mg·week^−1^), whereas those with any identifiable intake were defined as having reported caffeine exposure (>0 mg·week^−1^). This classification was used only to distinguish the absence versus presence of exposure within the assessment window and was not intended to represent a physiological threshold for caffeine habituation or tolerance. Therefore, occasional intake was not interpreted as established habitual caffeine use, and the primary inference remained based on the continuous exposure model.

To improve consistency in coding free-text responses, product information, and consumption-frequency formats, an AI-assisted standardized information-extraction procedure was implemented after completion of the experiment [32,33]. ChatGPT (GPT-5.5; OpenAI, San Francisco, CA, USA) was used from 2 July to 5 July 2026, solely for the structured extraction, organization, and standardization of questionnaire-derived information. All 30 participant records were processed using the same predefined prompt, fixed participant-level input template, coding rules, and standardized output structure. Before AI processing, all direct personal identifiers were removed, and only the study code and information required for caffeine-exposure processing were retained, including questionnaire-derived intake information, body-mass data, and researcher-verified caffeine reference information. The AI was explicitly instructed not to independently infer unknown product identities, serving sizes, consumption frequencies, or caffeine-content values, and any information that could not be reliably confirmed was uniformly flagged for manual review. The complete standardized prompt and participant-level input template are provided in Appendix A.

All 30 AI-assisted participant-level outputs were independently verified by two researchers against the original questionnaires and corresponding reference information before inclusion in the analytical dataset. The two researchers showed initial agreement for 28 of 30 records (93.3%). Initial disagreement occurred for 2 records (6.7%), both involving the correspondence between reported tea or tea-leaf consumption patterns and standardized product entries. These records were re-examined against the original questionnaire and relevant product information and were manually corrected, after which the two researchers reached agreement. The verification procedure prespecified that any disagreement remaining unresolved after rechecking and discussion would be adjudicated by a third researcher with relevant expertise; however, third-party adjudication was not required because both discrepancies were resolved by consensus. AI-generated outputs were used only as an intermediate standardized coding aid, and final product identification, caffeine-content attribution, frequency conversion, and participant-level caffeine-exposure estimation were based on the researcher-verified records.

### 2.5. Caffeine and Placebo Intervention

Individualized caffeine doses were calculated based on each participant’s body mass measured during the DXA assessment. In the CAF condition, participants ingested caffeine at a dose of 3 mg·kg^−1^ body mass using MuscleTech 100% Caffeine (MuscleTech^®^, Iovate Health Sciences International Inc., Oakville, ON, Canada). The commercial product used in this study contained a manufacturer-declared caffeine content of 200 mg per capsule. Throughout the study, all caffeine used to prepare the CAF condition supplements was obtained from the same commercial product batch. Supplement preparation was performed by an independent researcher who was not involved in participant testing, on-site data collection, or primary statistical analyses. For each participant, the individualized target caffeine dose of 3 mg·kg^−1^ was first calculated according to body mass. The original commercial caffeine capsules were then opened, and the amount required to achieve the participant-specific target dose was individually weighed using a precision electronic balance (Sanliang AB303, Dongguan Sanliang Measuring Tools Co., Ltd., Dongguan, China; readability: 0.001 g [1 mg]) and subsequently transferred into identical standardized empty capsule shells. No separate numerical tolerance range for dose deviation was prospectively specified; rather, each individualized preparation was weighed as closely as possible to the calculated target dose within the readability of the balance. The same product batch and standardized preparation procedure were used throughout the study to minimize variation in supplement preparation across experimental sessions, and individualized dosing was based on the manufacturer-declared caffeine content of the commercial product. In the PLA condition, food-grade starch was filled into the same type of capsule shells. CAF and PLA capsules were identical in appearance, capsule size, packaging, labeling, and administration procedures. Each participant’s supplements were labeled and allocated according to a pre-generated randomization code, which was retained by the independent researcher responsible for supplement preparation. Throughout data collection, participants and on-site testing personnel remained unaware of the assigned treatment condition, and the randomization code was not revealed until all experimental testing and prespecified primary data-processing procedures had been completed. All supplement ingestion was completed under the direct supervision of research staff, and complete ingestion of the assigned capsules was confirmed before the standardized 60-min absorption period. Accordingly, supplement-ingestion compliance was 100% for all experimental sessions that were initiated. Participants were also monitored during the experiments for subjective symptoms or discomfort potentially associated with supplementation. Three self-reported events were documented. One participant reported a subjective sensation of an accelerated heartbeat during one experimental session. Concurrent real-time heart-rate monitoring using a Polar V800 heart rate monitoring system (Polar Electro Oy, Kempele, Finland) did not indicate an obvious abnormal increase. After reassessment, the participant reported feeling well and was considered able to safely continue the experiment; therefore, the session was completed without interruption. After unblinding at the end of the study, this event was found to have occurred during the PLA condition. In addition, two participants reported a marked delay in sleep onset on the night following the CAF condition and remained awake until approximately 04:00–05:00. None of these events resulted in medical intervention, termination of an experimental session, or participant withdrawal, and no serious adverse events were observed during the study.

### 2.6. Standardized Warm-Up

At the beginning of each experimental trial, participants’ resting heart rate (HR) and rating of perceived exertion (RPE) were recorded, followed by a standardized warm-up protocol. The warm-up commenced with 5 min of low-intensity jogging, during which heart rate was maintained at approximately 120 beats·min^−1^. Participants then performed a series of dynamic mobility exercises, including knee-to-chest calf raises, plantar circular movements, and the quadruped squat combined with diagonal leg reach, with 8 repetitions per side for 2 sets. Subsequently, participants completed the World’s Greatest Stretch, performing 3 repetitions per side for 2 sets. Between sets, participants returned to the starting position by slow walking, which was considered active recovery between sets. All participants completed the identical warm-up protocol, exercise sequence, and training volume in both the CAF and PLA trials. Following completion of the warm-up, HR and RPE were recorded again, and participants performed the pre-conditioning activity countermovement jump (CMJ) assessment. The CMJ performance obtained after the warm-up and before the conditioning activity was defined as the Pre-CA baseline value, which was used to evaluate the subsequent temporal response following individualized DJ-induced PAPE.

### 2.7. Individualized DJ Conditioning Activity Protocol

Following completion of the familiarization procedures and before the formal crossover trials, all participants completed a separate standardized multi-height drop jump (DJ) assessment to determine the individualized optimal drop height (ODH) subsequently used for the conditioning activity. Participants performed DJs from 20, 30, 40, and 50 cm in a fixed ascending sequence, completing three valid maximal-effort trials at each height. Approximately 15 s of recovery was allowed between successive trials at the same height, providing sufficient time for the participant to return to the starting position and prepare for the next maximal-effort attempt. A 1-min recovery period was provided between successive drop heights. For each trial, participants were instructed to minimize ground contact time after landing while performing a maximal vertical jump, with ground contact time constrained to ≤250 ms to emphasize fast stretch–shortening cycle characteristics [34].

DJ performance during this assessment was measured using a Kunwei force-platform system (Kunwei Technology Co., Ltd., Shanghai, China) at a sampling frequency of 1000 Hz. Jump height and ground contact time were obtained from the force-platform system, and the reactive strength index (RSI) was automatically calculated by the accompanying software according to the following equation [35]:

RSI = Jump height/Ground contact time (1)

For each drop height, the highest RSI obtained from the three valid trials was retained. The highest RSI across the four tested heights was subsequently identified as the participant’s peak RSI, and the corresponding drop height was designated as the individualized ODH [7]. This ODH was then fixed and used during both the CAF and PLA experimental trials. The multi-height assessment was conducted specifically as an individualized prescription-setting procedure for the subsequent conditioning activity rather than as an experimental outcome itself. Accordingly, the peak RSI and its corresponding ODH were retained for each participant to determine the subsequent conditioning prescription. This approach was selected on the basis of previous evidence supporting the reliability of peak RSI and ODH determination using multi-height DJ protocols [7]. In comparable multi-height testing, good interday reliability has been reported for peak RSI (ICC = 0.87; CV = 4.20%) and ODH (ICC = 0.81; CV = 2.98%) [7].

During each experimental trial, participants ingested the assigned CAF or PLA supplement 60 min before the subsequent performance assessments and completed the standardized waiting period, warm-up, and Pre-CA assessment. Supplementation timing and experimental procedures were identical between the two crossover conditions.

After completion of the Pre-CA assessment, participants performed the conditioning activity from their previously determined individualized ODH. The same participant-specific ODH was used in both CAF and PLA trials. The conditioning activity consisted of 3 sets of 5 DJ repetitions, with 1 min of recovery between sets. All repetitions were performed according to standardized technical instructions and were supervised by trained researchers to maintain consistency in the prescribed conditioning procedure. Because the formal 3 × 5 DJ protocol served as the conditioning activity itself rather than as a separate biomechanical assessment, repetition-level RSI, ground contact time, and achieved jump height were not recorded during the CAF and PLA conditioning bouts. Consequently, the realized mechanical characteristics of the conditioning DJs could not be compared directly between conditions.

### 2.8. Temporal Assessment of PAPE Response

Following the individualized DJ conditioning activity, countermovement jump (CMJ) performance was assessed at Pre-CA, 15 s, 3 min, 6 min, 9 min, and 12 min to characterize the temporal trajectory of performance changes after the conditioning stimulus. The nominal post-conditioning labels refer to the targeted assessment windows used consistently throughout the protocol. At each time point, two duplicate maximal-effort CMJs were performed sequentially, with approximately 15 s between trials to allow repositioning and preparation for the second effort. For the nominal 15-s assessment, the first CMJ was initiated within the targeted early post-conditioning window and the second was completed after the same brief standardized inter-trial recovery.

CMJ was measured using a KWYP-FP6035-A2 one-dimensional force-platform system (Kunwei Technology Co., Ltd., Shanghai, China) at a sampling frequency of 1000 Hz. Participants maintained their hands on their hips to minimize the influence of arm swing and performed maximal-effort CMJs according to standardized technical instructions. Vertical force-time data were processed by the accompanying Kunwei software (KW3.0.10.9M, Kunwei, Shanghai, China), and jump height was obtained from the software-derived output. A closely corresponding Kunwei KWYP-FP6035 system has previously demonstrated concurrent validity against a Kistler 9287CA in-ground force plate during CMJ testing, with no fixed or proportional bias across common CMJ variables and ICC values > 0.950; the validation protocol also sampled at 1000 Hz and calculated jump height using the take-off-velocity/impulse-momentum approach [36].

Two valid CMJ trials were completed at each time point, and the highest jump height was retained as the prespecified representative value. This best-of-two rule was applied identically across all conditions and time points.

To quantify study-specific within-session repeatability of the duplicate CMJ measurements, the typical error (TE), coefficient of variation (CV), and a two-way mixed-effects, absolute-agreement, single-measure intraclass correlation coefficient [ICC(A,1)] were calculated from the paired Trial 1 and Trial 2 measurements. TE was calculated as:

TE = SD(Trial 2 − Trial 1)/√2(2)

CV was calculated relative to the grand mean of the two duplicate trials:

CV (%) = 100 × TE/Grand mean of Trial 1 and Trial 2(3)

Across the post-conditioning time points (15 s–12 min), CVs ranged from 2.02% to 3.03% under PLA and from 1.73% to 3.59% under CAF; corresponding ICC(A,1) values ranged from 0.946 to 0.977 and from 0.933 to 0.983, respectively. Detailed trial-level descriptive statistics, TE, CV, and ICC values are provided in Table A2. The smallest worthwhile change (SWC) for CMJ was calculated separately as 0.2× the between-participant SD of participant-level mean Pre-CA CMJ and was used only as a contextual benchmark rather than an equivalence margin.

### 2.9. Heart Rate and Rating of Perceived Exertion

Heart rate (HR) and rating of perceived exertion (RPE) were included as secondary outcomes to characterize the physiological and perceptual temporal responses under the CAF and PLA conditions. HR was monitored and recorded at rest, during the warm-up jogging period, after completion of the warm-up, before the conditioning activity (Pre-CA), and at the corresponding post-conditioning time points. RPE was assessed at rest, after the warm-up, at Pre-CA, and at the corresponding post-conditioning time points.

HR was continuously monitored using the Polar V800 heart rate monitoring system (Polar Electro Oy, Kempele, Finland). During testing, participants wore the corresponding chest-strap heart rate sensor, which was positioned securely at the lower edge of the chest and maintained stable contact with the skin to ensure continuous and reliable signal acquisition during exercise. During the warm-up jogging phase, exercise intensity was controlled at approximately 120 beats·min^−1^ through real-time HR monitoring.

RPE was assessed using the Borg CR10 scale, with participants providing ratings based on their overall perceived exertion during testing. In the statistical analyses, HR and RPE were analyzed as secondary outcomes, and both absolute values at each time point and changes relative to Pre-CA were considered when applicable.

### 2.10. Statistical Analysis

Continuous variables were summarized as mean ± SD or median [interquartile range], as appropriate, and categorical variables as *n* (%). Normality was assessed using the Shapiro–Wilk test. Descriptive comparisons of continuous participant characteristics between reported-caffeine-exposure groups used independent-samples *t* tests or Mann–Whitney U tests as appropriate, with Cohen’s d or rank-biserial correlation reported as effect sizes.

Sample-size planning was performed a priori before participant recruitment and was aligned with the primary Condition × Time hypothesis. Because the principal question concerned whether the within-participant CAF–PLA difference in CMJ varied across the five post-conditioning time points (15 s, 3, 6, 9, and 12 min), the calculation was formulated in terms of the repeated temporal profile of these paired condition differences. The prespecified expected CAF–PLA differences across the five time points were 1.50, 1.00, 0.50, 0.20, and 0.00 cm, respectively, with an assumed SD of 2.5 cm for the paired condition differences, a correlation of 0.50 among repeated difference scores, and a nonsphericity correction of ε = 0.75. With α = 0.05 and 80% target power, simulation-based repeated-measures power analysis indicated that 30 participants were required to detect the prespecified temporal Condition × Time pattern. The estimated power at *n* = 30 was approximately 0.82. Accordingly, 30 participants were recruited and randomized before the crossover trial commenced.

The primary analysis included all valid observations contributed by randomized participants. Post-conditioning countermovement-jump (CMJ) height at 15 s, 3, 6, 9, and 12 min was analyzed using a restricted maximum-likelihood linear mixed-effects model with Condition, Time, Condition × Time, McKay tier, Period, and Sequence as fixed effects; Time was modeled categorically. Random intercepts were specified for participants and sessions nested within participants, with a continuous-time first-order autoregressive [CAR(1)] residual correlation structure within sessions. The global Condition × Time interaction was the primary inferential test. Adjusted marginal means, 95% confidence intervals, and CAF–PLA contrasts were estimated using emmeans; the five time-specific primary contrasts were Holm-adjusted, and standardized effects were calculated as the contrast estimate divided by the model residual SD. Model-based degrees of freedom were reported, with containment degrees of freedom used for estimated marginal means and contrasts.

Pre-CA CMJ, measured after supplement ingestion and before the conditioning activity, was analyzed separately and was not included as a covariate in the primary model. Sensitivity analyses additionally adjusted for Pre-CA CMJ, standardized DXA-derived lean mass and body-fat percentage, or restricted the analysis to complete cases. A corresponding model without the CAR(1) structure was fitted for covariance-structure comparison. Model diagnostics comprised normalized residual-versus-fitted plots, normal Q–Q plots, residual autocorrelation functions, screening of absolute normalized residuals >3, and participant-level leave-one-out analyses.

ΔCMJ was analyzed as a supportive outcome using the same longitudinal mixed-effects structure. Habitual caffeine exposure was examined exploratorily. Continuous habitual caffeine intake (mg·kg^−1^·day^−1^) was transformed using log(1 + x), z-standardized, and entered in a Condition × Time × Exposure model; contrasts were estimated at zero exposure, the median non-zero exposure, and the 90th percentile, with Holm adjustment across 15 contrasts. A secondary exploratory model used binary reported caffeine exposure and tested Condition × Time × Group, with Holm adjustment across 10 time-by-group contrasts. Global three-way interactions were considered before time-specific contrasts.

Exploratory derived outcomes included maximum observed ΔCMJ, 0–12 min ΔCMJ area under the curve calculated by trapezoidal integration, and time to maximum observed ΔCMJ. Paired CAF–PLA comparisons used paired *t* tests when paired differences were normally distributed and Wilcoxon signed-rank tests otherwise; time to maximum observed ΔCMJ was analyzed with the paired Wilcoxon signed-rank test. Holm adjustment was applied across the three derived-outcome tests. Heart rate and rating of perceived exertion (RPE) were analyzed as secondary outcomes using analogous mixed-effects models with adjustment for the corresponding pre-conditioning value; the two global Condition × Time tests were Holm-adjusted as a secondary-outcome family.

The smallest worthwhile change for CMJ was calculated as 0.2 × the between-participant SD of participant-level mean Pre-CA CMJ and was used only as a contextual benchmark, not as an equivalence margin. Analyses were conducted in R version 4.3.2 (R Foundation for Statistical Computing, Vienna, Austria) using the nlme and emmeans packages. Two-sided *p* values and 95% confidence intervals are reported.

## 3. Results

### 3.1. Analysis Sample and Pre-CA CMJ

**Figure 3:**
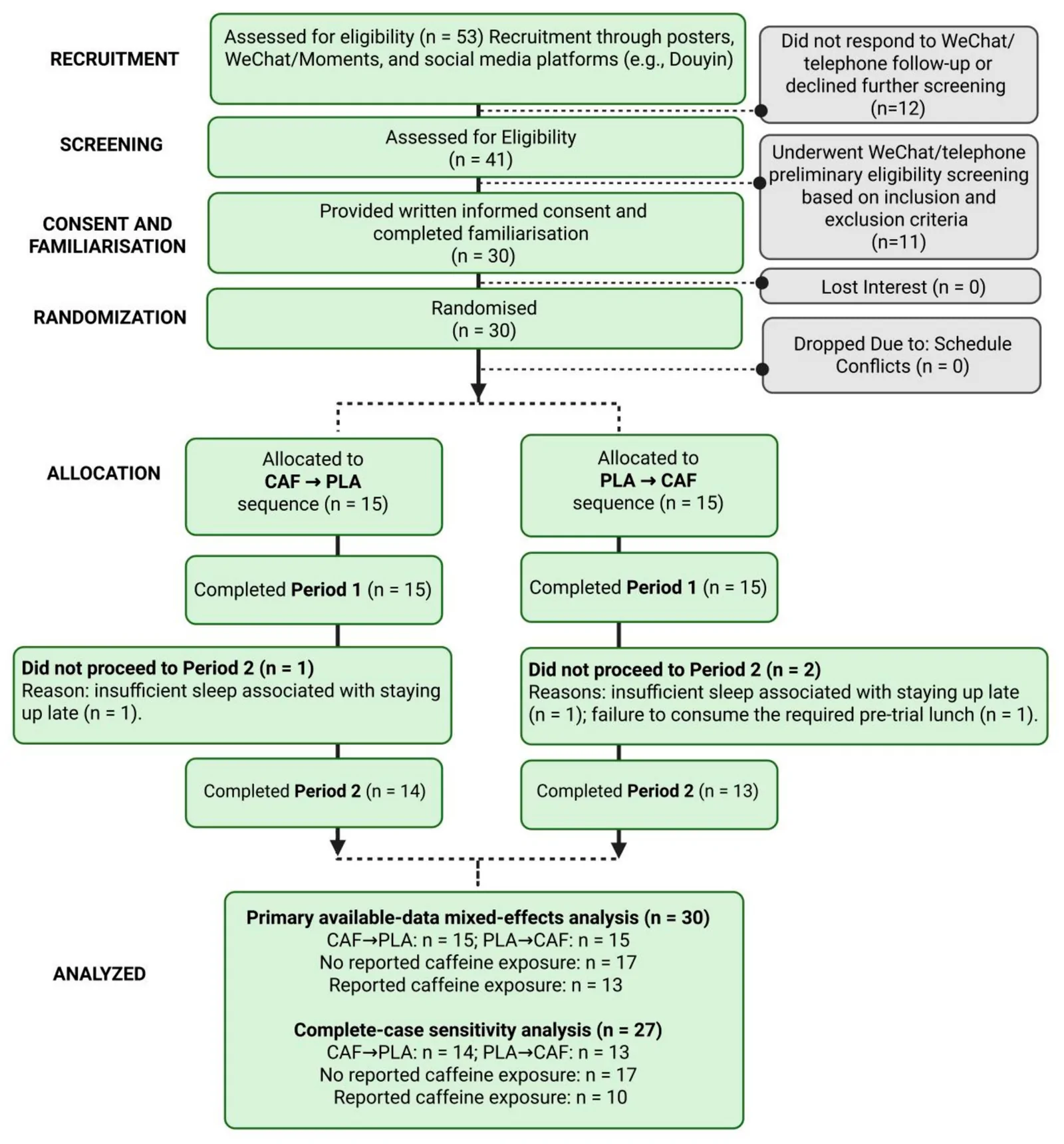
CONSORT flow diagram of recruitment, randomization, crossover completion, and analysis. CAF, caffeine; PLA, placebo.

All 30 randomized participants contributed data from at least one valid experimental period and were therefore included in the available-data mixed-effects analysis (Figure 3). Twenty-seven participants completed both crossover periods, whereas three did not proceed to Period 2 because predefined pre-trial readiness requirements were not met; their valid Period 1 data were retained. The available dataset therefore comprised 57 valid participant-period observations and 285 post-conditioning CMJ measurements. Fifteen participants were randomized to each sequence (CAF→PLA and PLA→CAF); among the 27 participants completing both periods, 14 followed CAF→PLA and 13 followed PLA→CAF.

Among the 30 participants included in the available-data analysis, 13 (43.3%) were classified as McKay Tier 2 and 17 (56.7%) as Tier 3. Seventeen participants (56.7%) reported no caffeine exposure during the assessment window and 13 (43.3%) reported caffeine exposure; the corresponding numbers among the 27 complete cases were 17 and 10. Participant characteristics for the randomized available-data and complete-case cohorts are presented in Table 1.

For all experimental periods that proceeded to formal testing, supplement ingestion was completed under direct investigator supervision, corresponding to 100% ingestion compliance among initiated sessions. Three self-reported adverse events were recorded. One participant reported a transient subjective perception of a faster heartbeat during PLA; no obvious abnormality was observed on concurrent Polar heart-rate monitoring, and the session was completed. Two participants reported delayed sleep, remaining awake until approximately 04:00–05:00. None of these events required medical intervention, session termination, or study withdrawal, and no serious adverse events occurred.

Pre-CA CMJ was measured after supplement ingestion and before the conditioning activity. The model-adjusted mean was 45.65 cm (95% CI 43.61–47.70) under PLA and 46.10 cm (95% CI 44.04–48.16) under CAF. The adjusted CAF–PLA difference was 0.45 cm (95% CI −0.58 to 1.48; F(1,25) = 0.80, *p* = 0.380), with no statistically clear between-condition difference at this measurement point (Table 2).

### 3.2. Primary Post-Conditioning CMJ Response

**Figure 4:**
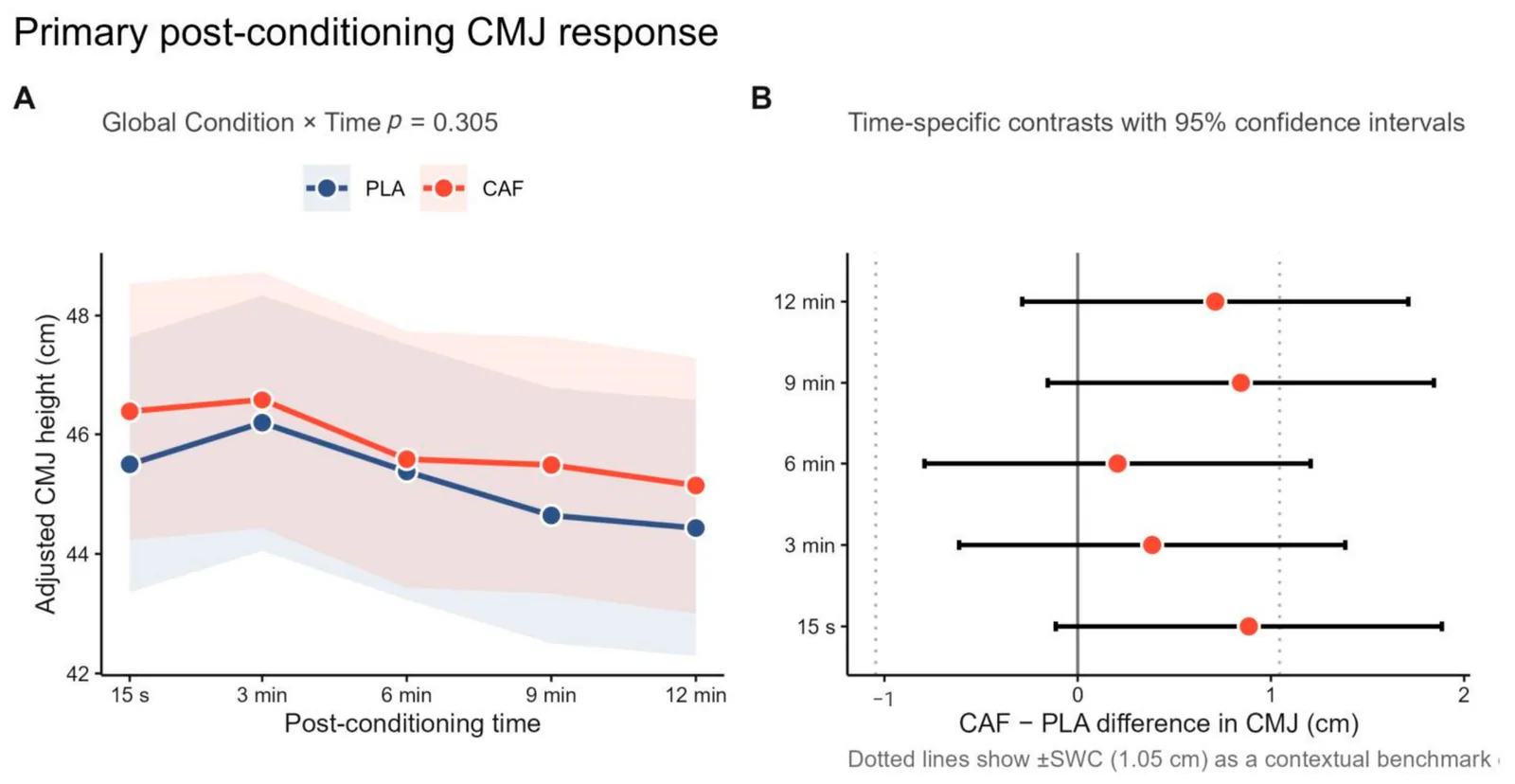
Primary post-conditioning CMJ response. (**A**) Adjusted CMJ means (95% CI) under PLA and CAF across five post-conditioning time points. (**B**) CAF–PLA contrasts (95% CI); the solid line denotes zero and dotted lines denote the ±1.05-cm SWC. CMJ, countermovement jump; CAF, caffeine; PLA, placebo.

In the primary mixed-effects model, CMJ performance varied across post-conditioning time (Time: F(4, 220) = 7.71, *p* < 0.001). The Condition effect did not reach statistical significance (F(1, 25) = 3.33, *p* = 0.080), and the primary Condition × Time interaction was also not significant (F(4, 220) = 1.22, *p* = 0.305) (Table 3; Figure 4). Model-adjusted CMJ height increased from 15 s to 3 min under both conditions and subsequently declined.

Estimated CAF–PLA differences were 0.89 cm (95% CI −0.11 to 1.88) at 15 s, 0.39 cm (−0.61 to 1.38) at 3 min, 0.21 cm (−0.79 to 1.21) at 6 min, 0.84 cm (−0.15 to 1.84) at 9 min, and 0.71 cm (−0.29 to 1.71) at 12 min. All five 95% CIs crossed zero, and none of the time-specific contrasts were significant after Holm adjustment (all Holm-adjusted *p* ≥ 0.399). Standardized condition differences ranged from 0.14 to 0.61 residual SDs. The post hoc SWC was 1.05 cm; all point estimates were below this benchmark, although the confidence intervals did not exclude effects exceeding it. The SWC was used only as a contextual benchmark and not as an equivalence margin.

### 3.3. Exploratory Caffeine-Exposure Moderation

**Figure 5:**
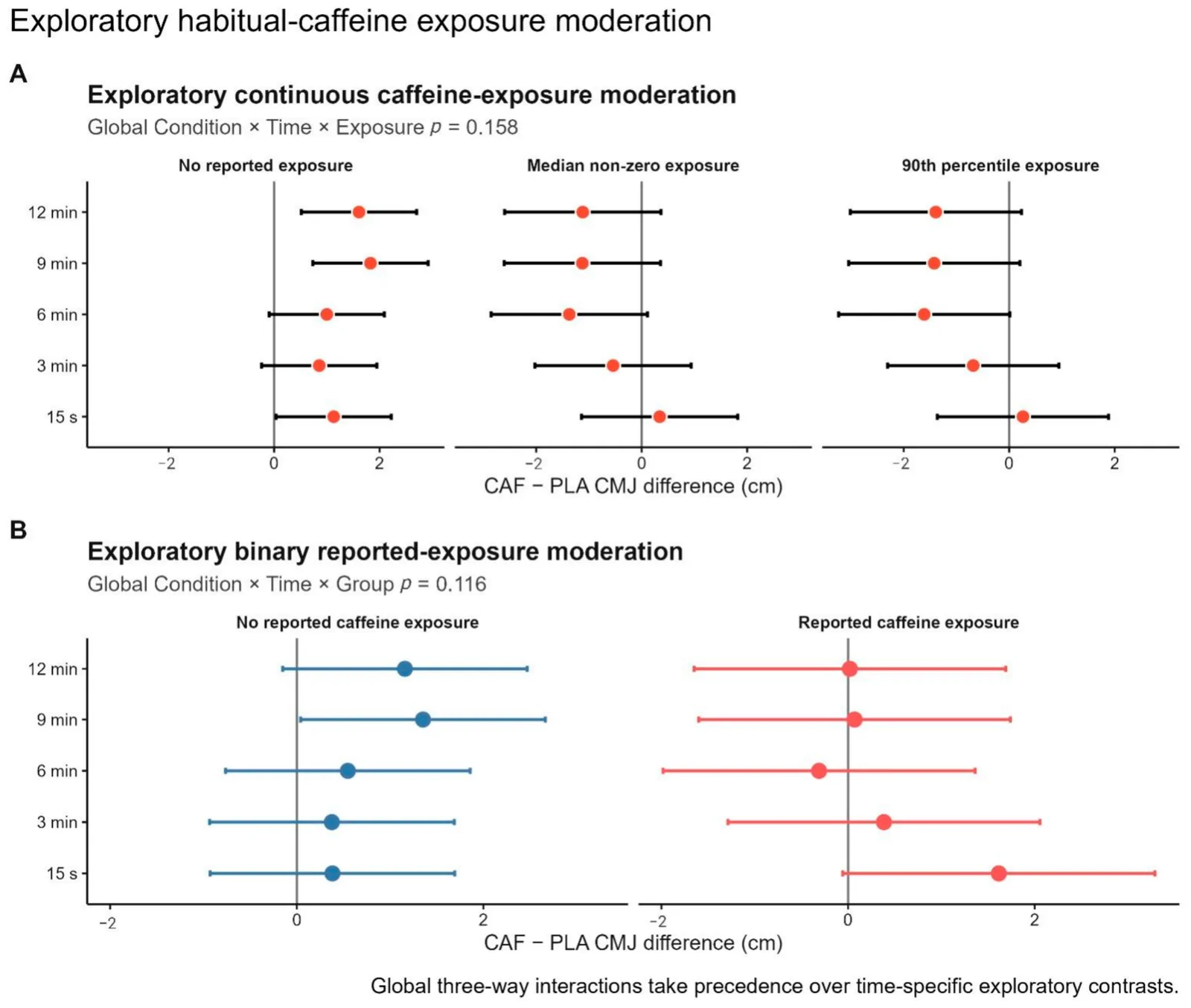
Exploratory caffeine-exposure moderation. (**A**) CAF–PLA CMJ contrasts at zero, median non-zero (0.80 mg·kg^−1^·day^−1^), and 90th-percentile (0.91 mg·kg^−1^·day^−1^) continuous exposure. (**B**) CAF–PLA contrasts by binary reported-exposure group. Points are estimates and bars are 95% CIs. CMJ, countermovement jump; CAF, caffeine; PLA, placebo.

Neither exploratory moderation model showed a statistically significant global three-way interaction. The Condition × Time × Exposure interaction was F(4, 212) = 1.67 (*p* = 0.158) in the continuous-exposure model, and the Condition × Time × Group interaction was F(4, 212) = 1.87 (*p* = 0.116) in the binary reported-exposure model (Table 4; Figure 5).

At the prespecified representative levels of continuous exposure, the only time-specific contrast that remained significant after Holm adjustment across all 15 comparisons occurred at 9 min at zero reported exposure (CAF–PLA = 1.83 cm, 95% CI 0.74–2.92; Holm-adjusted *p* = 0.031). All remaining continuous-exposure contrasts had Holm-adjusted *p* ≥ 0.078. No time-specific contrast in the binary model remained significant after adjustment (all Holm-adjusted *p* ≥ 0.435). Because neither global three-way interaction was significant, these time-specific patterns were treated as exploratory and hypothesis-generating.

### 3.4. Sensitivity and Covariance-Structure Analyses

**Figure 6:**
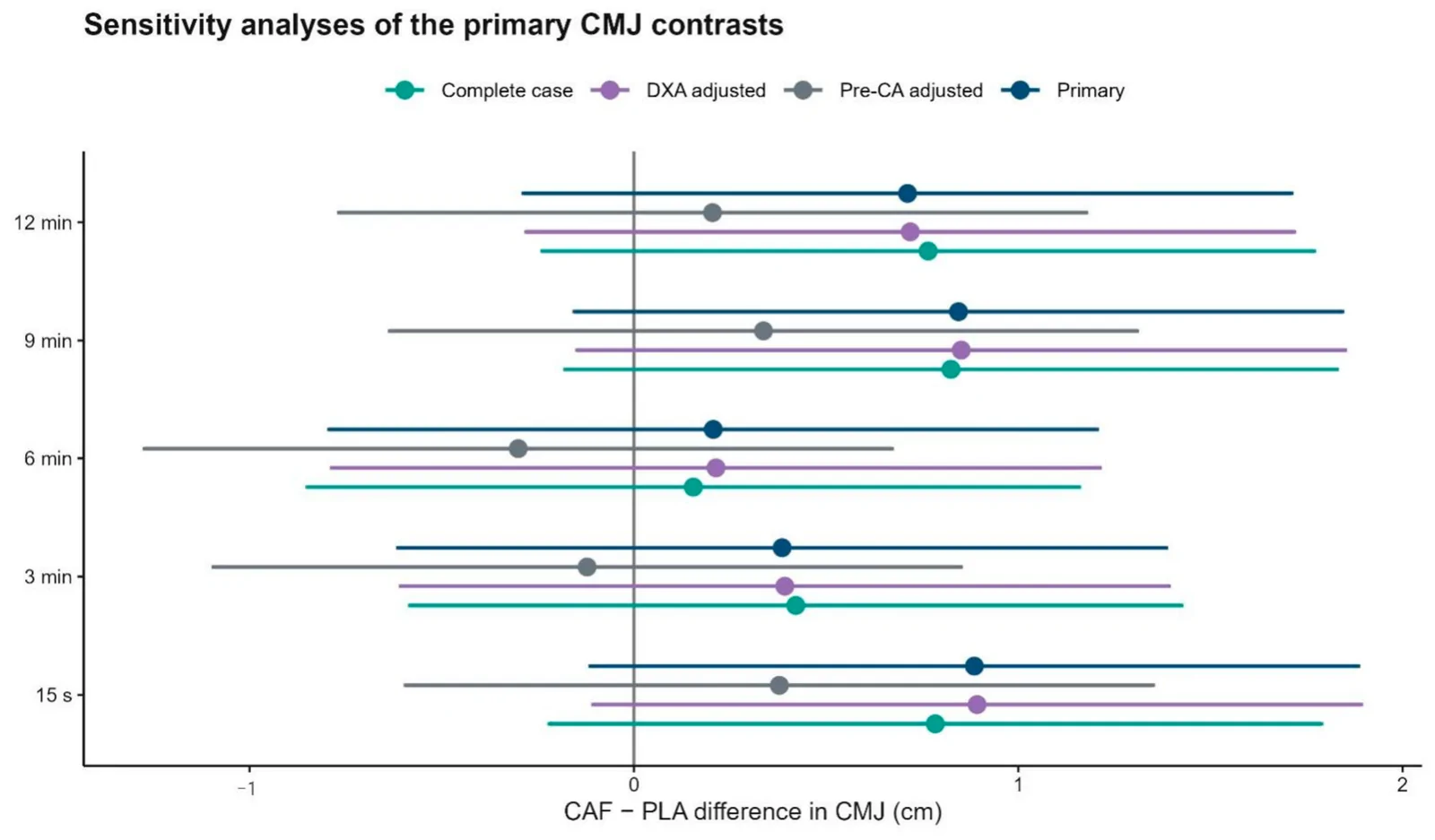
Sensitivity analyses of the primary CMJ contrasts. CAF–PLA estimates (95% CI) are shown for the primary, Pre-CA-adjusted, DXA-adjusted, and complete-case models; the vertical line denotes zero. CMJ, countermovement jump; CAF, caffeine; PLA, placebo; DXA, dual-energy X-ray absorptiometry.

The primary inference was consistent across sensitivity analyses. The Condition × Time interaction was F(4, 220) = 1.22 (*p* = 0.304) in the Pre-CA-adjusted model, F(4, 220) = 1.22 (*p* = 0.305) in the DXA-adjusted model, and F(4, 208) = 1.09 (*p* = 0.361) in the complete-case model (Table 5; Figure 6). Adjustment for Pre-CA attenuated the time-specific CAF–PLA estimates, whereas the DXA-adjusted and complete-case estimates remained close to those from the primary available-data model; all sensitivity-model CIs crossed zero.

The nested random-intercept model with continuous-time AR(1) residual correlation had lower information criteria than the corresponding model without AR(1) (AIC 1093.95 vs. 1129.42; BIC 1155.25 vs. 1187.11) (Table 6).

### 3.5. Supportive ΔCMJ Analysis

**Figure 7:**
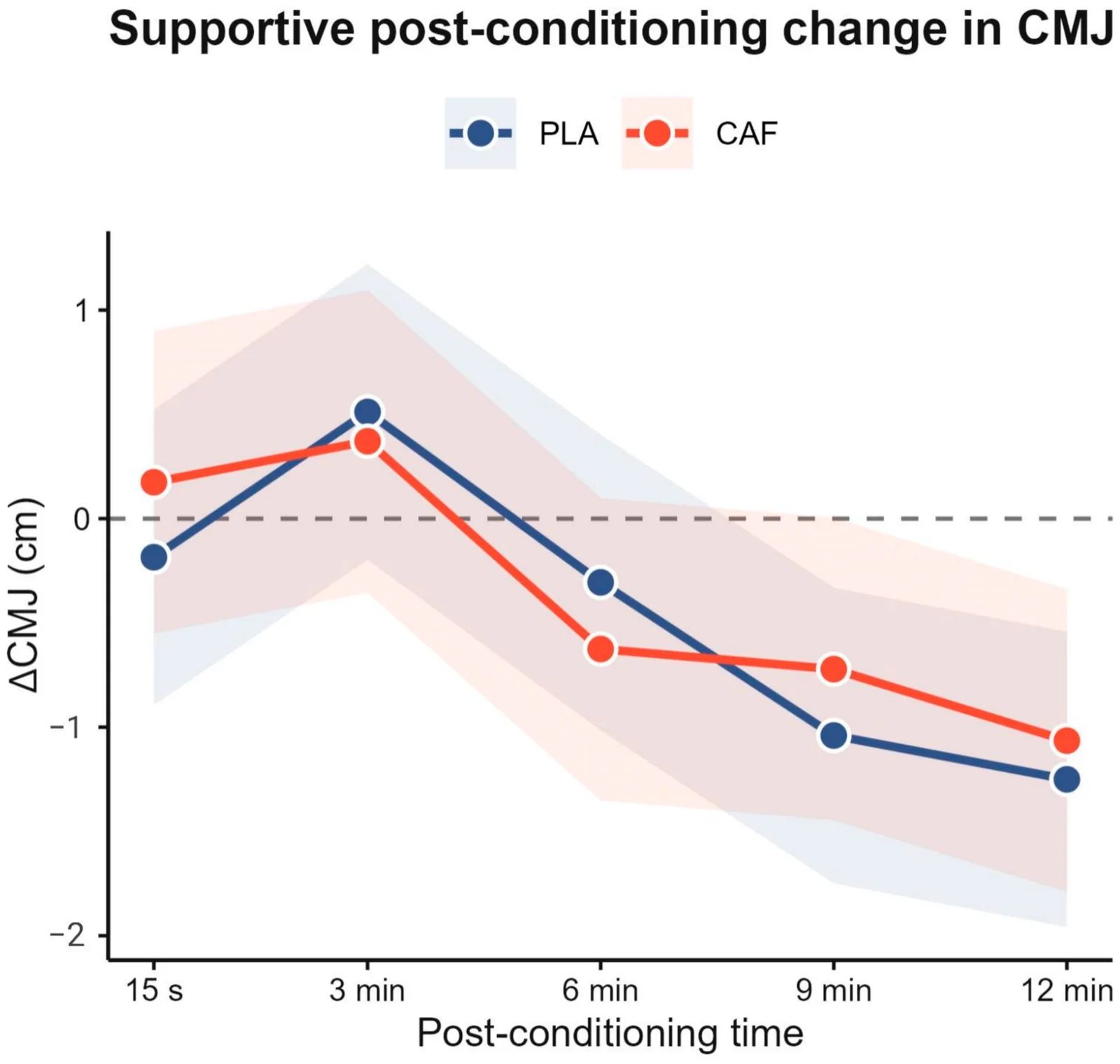
Supportive post-conditioning ΔCMJ response. Adjusted ΔCMJ trajectories under PLA and CAF are shown across five time points; shaded bands are 95% CIs and the dashed line denotes ΔCMJ = 0. ΔCMJ was calculated relative to post-supplement Pre-CA. CAF, caffeine; PLA, placebo.

The supportive ΔCMJ model showed a significant effect of Time (F(4, 220) = 7.68, *p* < 0.001), whereas neither Condition (F(1, 25) = 0.57, *p* = 0.456) nor Condition × Time (F(4, 220) = 1.22, *p* = 0.304) was significant (Table 7; Figure 7). Adjusted ΔCMJ values were slightly above zero at 3 min under both conditions and became negative thereafter, with larger negative changes at 9 and 12 min. All time-specific CAF–PLA confidence intervals crossed zero, and all Holm-adjusted *p* values were 1.000.

### 3.6. Exploratory Derived CMJ Outcomes

Among the 27 participants with paired data under both conditions, none of the three exploratory derived outcomes differed significantly between CAF and PLA after within-family Holm adjustment (Table 8). Mean maximum observed ΔCMJ was 1.06 cm under CAF and 0.78 cm under PLA (Wilcoxon signed-rank *p* = 0.962). The 0–12 min ΔCMJ AUC was −4.34 cm·min under CAF and −6.17 cm·min under PLA, with a paired mean difference of 1.82 cm·min (95% CI −7.95 to 11.60; *p* = 0.704; Cohen’s dz = 0.07). Mean time to maximum observed ΔCMJ was 3.77 min under CAF and 2.98 min under PLA (paired Wilcoxon *p* = 0.442). All three Holm-adjusted *p* values were 1.000. Across all valid condition-specific sessions, the most frequent time to maximum observed ΔCMJ was 3 min under PLA (12/29, 41.4%) and 15 s under CAF (12/28, 42.9%). This distribution is descriptive; paired inference was based only on participants completing both conditions.

### 3.7. Secondary HR and RPE Responses

**Figure 8:**
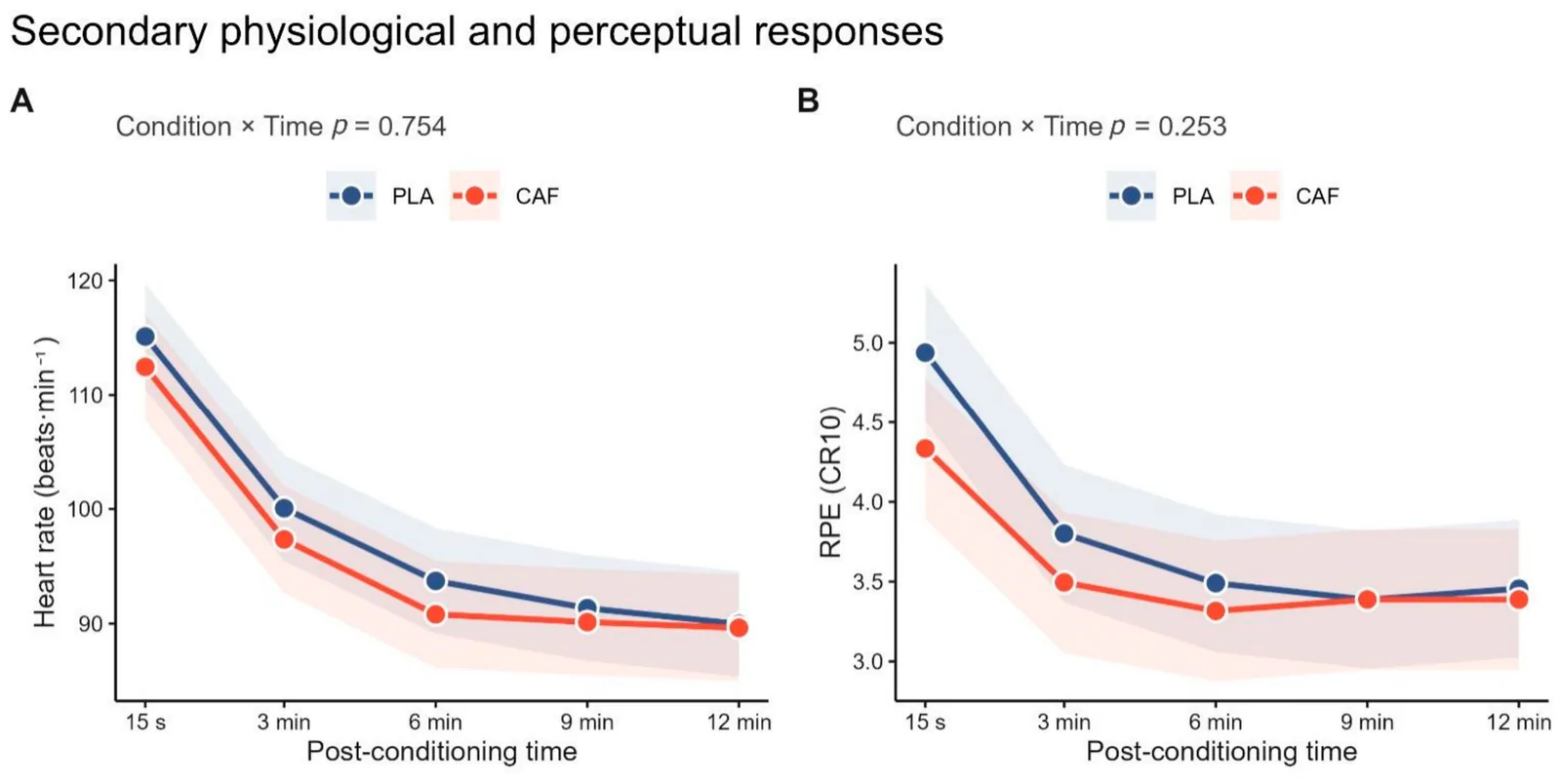
Secondary physiological and perceptual responses. (**A**) Adjusted HR trajectories under PLA and CAF. (**B**) Adjusted RPE trajectories under PLA and CAF. Shaded bands are 95% CIs. RPE, rating of perceived exertion; CAF, caffeine; PLA, placebo.

The secondary HR model showed a significant effect of Time (F(4, 220) = 83.20, *p* < 0.001), whereas neither Condition (F(1, 24) = 1.78, *p* = 0.195) nor Condition × Time (F(4, 220) = 0.48, *p* = 0.754) was significant (Table 9; Figure 8). HR declined over time under both conditions, and none of the five time-specific CAF–PLA contrasts were significant after Holm adjustment (all Holm-adjusted *p* ≥ 0.778).

RPE also showed a significant effect of Time (F(4, 220) = 25.57, *p* < 0.001), and the Condition effect was significant (F(1, 24) = 6.51, *p* = 0.018); however, the Condition × Time interaction was not significant (F(4, 220) = 1.35, *p* = 0.253). At 15 s, the unadjusted CAF–PLA difference was −0.60 points (95% CI −1.09 to −0.12; *p* = 0.018), but this contrast was not significant after Holm adjustment (*p* = 0.088); all remaining RPE contrasts had Holm-adjusted *p* ≥ 0.837.

### 3.8. Model Diagnostics and Influence Analysis

**Figure A1:**
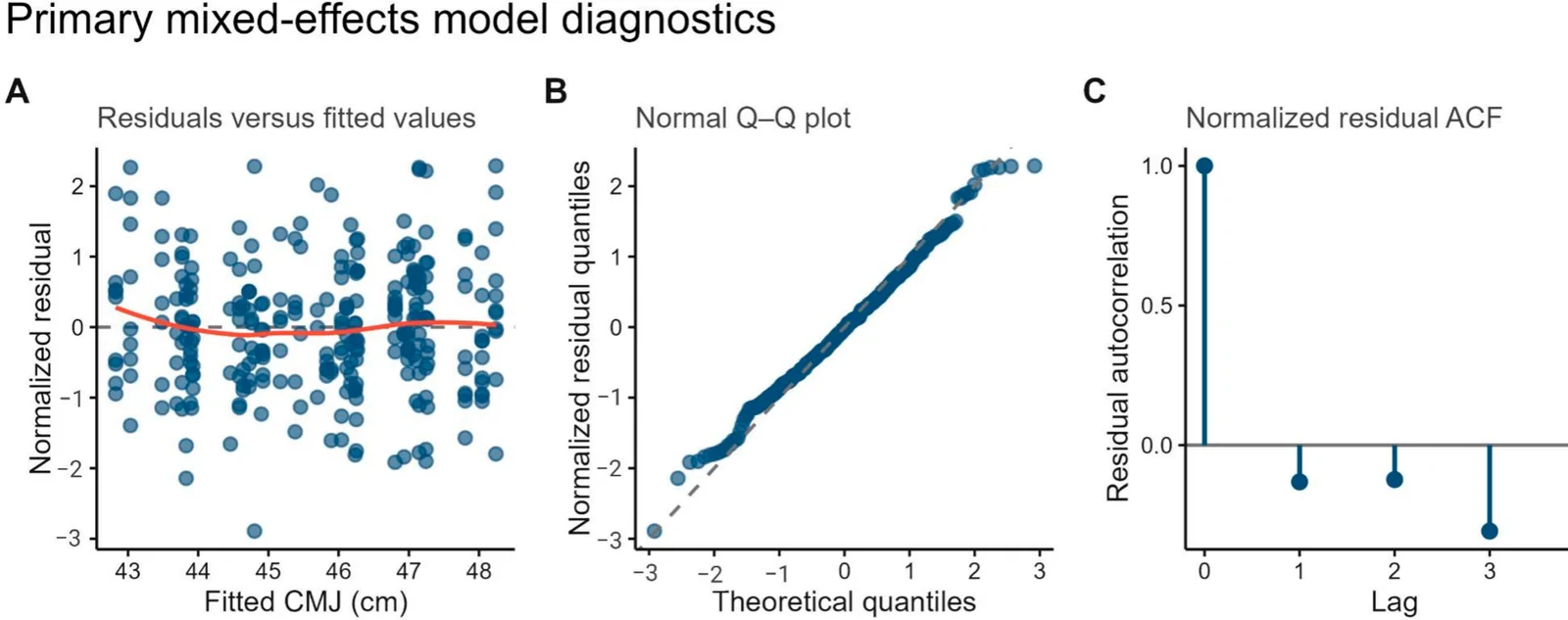
Primary mixed-effects model diagnostics. (**A**) Normalized residuals versus fitted CMJ values. (**B**) Normal Q–Q plot. (**C**) Residual autocorrelation after continuous-time AR(1) modeling. CMJ, countermovement jump.

Normalized residuals ranged from −2.89 to 2.29, with no observation exceeding |3|. All 30 leave-one-participant-out refits converged, and the primary Condition × Time *p* value ranged from 0.124 to 0.462. After model fitting, residual autocorrelations at lags 1–4 were −0.13, −0.12, −0.31, and −0.18, respectively. Overall diagnostics did not indicate that the primary inference was driven by any single participant (Figure A1).

## 4. Discussion

The present study employed a randomized, double-blind, placebo-controlled crossover design to examine whether acute ingestion of 3 mg·kg^−1^ caffeine could alter the temporal countermovement jump (CMJ) response following reactive strength index (RSI)-individualized drop-jump (DJ) conditioning in Chinese Han male basketball players, and further explored whether habitual caffeine exposure was involved in moderating this process. The main findings showed that CMJ performance changed significantly across post-conditioning recovery time, whereas the overall temporal trajectories under CAF and PLA did not show a statistically clear divergence. The primary Condition × Time interaction was not significant, and none of the CAF–PLA contrasts at the five prespecified time points remained significant after Holm adjustment. This overall inference remained consistent after adjustment for Pre-CA CMJ, further adjustment for DXA-derived lean mass and body-fat percentage, and restriction to complete cases in the sensitivity analyses; the supportive ΔCMJ analysis and derived outcomes, including maximum observed ΔCMJ, 0–12 min AUC, and time to maximum observed ΔCMJ, likewise showed no consistent advantage for CAF. In addition, no significant global three-way interaction was observed whether habitual caffeine exposure was modeled as a continuous variable or represented categorically. Accordingly, the present findings should not be simply summarized as “caffeine was ineffective”; a more accurate interpretation is that, when both conditions received the same RSI-individualized DJ conditioning stimulus, 3 mg·kg^−1^ caffeine did not provide sufficiently consistent evidence that it could further reshape the subsequent temporal CMJ response.

The significance of this finding becomes particularly apparent when it is placed within the existing “caffeine × PAPE” research framework, because the limited number of direct studies currently available have not yielded consistent conclusions. Guerra et al. administered 5 mg·kg^−1^ caffeine to 12 professional soccer players in combination with a conditioning stimulus comprising plyometric exercise and sled towing, and found that CMJ performance at 1, 3, and 5 min after conditioning was higher under CAF than under PLA, suggesting that caffeine might further amplify post-conditioning jump performance [37]. In contrast, Filip-Stachnik et al. administered 6 mg·kg^−1^ caffeine to semiprofessional female volleyball players in combination with an 80% 1RM velocity-controlled squat protocol; although improvements in CMJ were observed after the conditioning activity, CAF did not produce a clear additional benefit relative to PLA [12]. The overall findings of the present study are more consistent with the latter. Taken together, the existing evidence suggests that the independent ergogenic effect of caffeine does not necessarily translate into an additional enhancement of post-conditioning performance, and that the combined effect of CAF and a conditioning activity may depend more strongly on the context-specific matching of caffeine dose, conditioning-stimulus characteristics, performance task, and recovery time window.

This interpretation is also consistent with the basketball caffeine literature. A recent systematic review and meta-analysis of basketball players included 18 blinded crossover studies and showed that low-to-moderate doses of caffeine may produce small improvements in linear sprinting, repeated-sprint ability, agility, and single-jump performance; however, the pooled effect for single-jump performance itself was small (SMD approximately 0.15), indicating that the ergogenic effects of caffeine are not of equal magnitude across all basketball-related tasks [38]. Earlier, Abian-Vicen et al. also reported that approximately 3 mg·kg^−1^ caffeine produced a modest improvement in CMJ height in basketball players [39]. Therefore, although the present study did not observe a stable additional advantage of CAF, this does not negate the general ergogenic potential of 3 mg·kg^−1^ caffeine in basketball. Rather, it further delineates the boundary of this effect: when the comparison is no longer simply CAF versus PLA, but CAF and PLA superimposed on the same already individualized conditioning stimulus, the additional performance margin provided by caffeine may be substantially reduced.

This task dependence may also help explain the difference between a recent study in Chinese male collegiate basketball players and the present findings. Ma et al. used 5 mg·kg^−1^ caffeine combined with a complex PAPE protocol and found that, compared with PAPE + PLA, PAPE + CAF further increased Wingate peak power, mean power, and total work [13]. In contrast, our study used 3 mg·kg^−1^ caffeine, RSI-individualized DJ conditioning, and a single maximal CMJ as the primary performance outcome. Although the participant backgrounds in the two studies were broadly comparable, the principal physiological constraints of the performance tasks differed substantially. The 30-s Wingate test used by Ma et al. does not simply reflect instantaneous anaerobic power; rather, mechanical output must be sustained under an extremely high ATP turnover demand through the dynamic contribution of multiple energy systems. Classic quantitative work has shown that, over a complete 30-s Wingate test, approximately 56% of the energy demand is supplied by glycolysis, approximately 28% by the ATP–PCr system, and approximately 16% by oxidative metabolism, with the relative contribution of these systems shifting dynamically as exercise continues [40], alongside progressive phosphocreatine depletion and disruption of intramuscular energy homeostasis [41]. In contrast, the single maximal CMJ used in the present study is a very brief ballistic stretch–shortening-cycle task, whose performance depends more directly on the ability of the lower-limb neuromuscular system to generate effective force–time characteristics, positive impulse, and sufficient take-off velocity within a limited time, with comparatively less reliance on sustained metabolic energy provision [42,43]. Therefore, the difference in caffeine effects between the two studies may not simply reflect differences in the participant populations, but may instead reflect the matching between caffeine dose and task-specific physiological demands: through central neural modulation mediated by adenosine-receptor antagonism, and the consequent potential increases in motor drive and corticospinal excitability, caffeine may have greater temporal and physiological scope to translate into detectable performance benefits during a Wingate-type task in which metabolic and neuromuscular constraints progressively develop with sustained exercise [9,44], whereas the additional performance reserve that can be released during a single maximal CMJ following a sufficient warm-up and individualized conditioning stimulus may be comparatively limited.

Beyond average effects, the temporal trajectory observed in the present study also provides important information. Model-adjusted CMJ under both conditions increased from 15 s to approximately 3 min and then gradually declined, whereas supportive ΔCMJ values became more clearly negative at 9 and 12 min. Previous systematic reviews and meta-analyses have indicated that post-conditioning performance responses are not characterized by a simple unidirectional enhancement, but instead reflect the dynamic evolution of potentiation-related and fatigue-related processes over the recovery period. Seitz and Haff reported that recovery intervals of 3–7 min are generally more favorable for explosive performance [6], while Chen et al. further reported that approximately 4–9 min may, overall, constitute a relatively favorable window for jump performance [45]. Accordingly, the relatively higher CMJ observed around 3 min in the present study is directionally consistent with previous evidence regarding post-conditioning time windows. However, because the present study did not include a no-conditioning control, this temporal pattern cannot be directly interpreted as causal evidence of DJ-induced PAPE. More rigorously, the present study describes the CMJ recovery trajectory observed after completion of the same individualized DJ conditioning protocol, rather than independently quantifying the causal effect of the DJ conditioning itself.

On this basis, a more noteworthy finding is that caffeine did not provide evidence of systematically altering this temporal structure. Across all valid experimental periods, the maximum observed ΔCMJ most frequently occurred at 3 min under PLA and at 15 s under CAF; however, the formal paired analysis did not identify a condition difference in time to maximum observed ΔCMJ. Filip-Stachnik et al. similarly noted that CAF might lead some athletes to exhibit their best post-conditioning performance earlier, but the overall magnitude of improvement did not clearly differ between CAF and PLA, and substantial inter-individual variability in the optimal recovery time was evident [12]. Through repeated assessment at 15 s, 3, 6, 9, and 12 min, the present study further indicates that the available evidence is insufficient to support the conclusion that CAF shifts the optimal performance window earlier at the group level. Instead, a more important observation is that, even when drop height was individualized according to each athlete’s peak RSI, the time at which maximum subsequent performance occurred remained highly dispersed. This suggests that “individualization of the conditioning dose” and “individualization of recovery time” may represent two related but non-equivalent dimensions of prescription. RSI can help identify a more appropriate DJ height for an individual, but it does not ensure that different athletes will enter their optimal performance state at the same recovery time. Therefore, future individualization of PAPE practice may need to move beyond simply “giving each athlete a different stimulus” toward the joint individualization of both the conditioning stimulus and the recovery window.

Habitual caffeine exposure likewise did not provide a clear explanation for this response heterogeneity. Neither continuous exposure expressed in mg·kg^−1^·day^−1^ nor the binary representation of “no reported exposure/reported exposure” produced a significant global Condition × Time × Exposure interaction. This finding is consistent with part of the existing literature. Gonçalves et al. stratified endurance-trained men according to low, moderate, and high habitual caffeine intake and found no significant modification of the acute ergogenic effect of caffeine by habitual intake level [16]; Painelli et al. similarly reported that habitual caffeine consumption did not clearly attenuate the performance effects of acute supplementation in strength-endurance and jumping tasks [17]. However, the evidence in this area is not entirely consistent. Beaumont et al. observed an attenuation of the acute ergogenic effect of caffeine after 4 weeks of regular caffeine supplementation in individuals with low habitual intake, suggesting that some degree of tolerance may develop under specific doses and chronic exposure patterns [36]. Therefore, rather than summarizing the literature as indicating that “habitual users are insensitive to caffeine” or that “habitual intake has no effect”, a more reasonable interpretation may be that habitual exposure is only one of multiple moderators of the acute CAF response, and that its influence may be jointly constrained by habitual dose, exposure duration, acute supplementation dose, and the performance task.

Within this context, the approximately 1.83-cm CAF–PLA difference observed at 9 min at zero reported exposure should be interpreted within the overall evidential framework. Although this comparison remained statistically significant after Holm adjustment across the 15 prespecified contrasts, the global Condition × Time × Exposure interaction was not significant; therefore, this single time-point finding is insufficient to establish a stable pattern in which individuals with lower exposure respond more strongly to caffeine. In addition, the binary exposure indicator in the present study was used only to distinguish whether identifiable caffeine intake had been reported during the previous 4 weeks and was not based on a physiological threshold for caffeine habituation or tolerance; continuous exposure was the primary representation. Accordingly, this isolated signal is more appropriately regarded as hypothesis-generating rather than as a definitive subgroup effect. In light of the inconsistent findings in previous habitual-caffeine studies [16,17,36], future work may benefit from treating exposure as a continuum rather than simply categorizing athletes as “habitual/non-habitual,” and from jointly modeling habitual exposure dose, acute supplementation dose, abstinence status, and individual response.

The HR and RPE results provide additional context for the task specificity described above. HR declined across recovery time under both conditions, and CAF did not significantly alter the Condition × Time trajectory; although RPE showed a Condition effect, the Condition × Time interaction was not significant, and the CAF–PLA difference at 15 s was no longer significant after Holm adjustment. Previous studies have frequently reported that caffeine may reduce perceived effort or fatigue during exercise [9], but a perceptual change does not necessarily occur in parallel with an improvement in maximal explosive performance. The basketball meta-analysis likewise suggests that the magnitude of caffeine effects differs across performance and perceptual outcomes [38]. Thus, under the present protocol, RPE showed a certain condition-related signal without a concomitant statistically clear difference in the temporal CMJ trajectory. Because the present study did not directly test whether the treatment effects of CAF on RPE and CMJ differed from one another, the observation that one outcome was statistically significant while another was not should not be used to infer a so-called “perception–performance dissociation.”

From a practical perspective, the significance of the present findings is not to argue against caffeine use in basketball players, but rather to indicate that when pre-competition preparation already includes an RSI-individualized DJ protocol, an additional intake of 3 mg·kg^−1^ caffeine should not be assumed to further improve jump performance or prolong the effective performance window. The overall basketball literature still supports a small ergogenic effect of caffeine on selected physical-performance outcomes, but this effect appears to be strongly task- and context-dependent [38]. In addition, Stojanović et al. reported that ingestion of 3 mg·kg^−1^ caffeine in the morning improved selected jumping, sprinting, and change-of-direction outcomes in basketball players, whereas comparable benefits were not evident when caffeine was ingested in the evening, suggesting that time of ingestion may further influence the practical value of CAF [46]. All testing in the present study was conducted within a relatively standardized afternoon window, and two participants reported marked delayed sleep onset after CAF. Therefore, for late-afternoon or evening competition, evaluation of a caffeine strategy should not focus solely on its potential immediate ergogenic benefit, but should also consider possible subsequent sleep costs. In applied practice, whether CAF should be combined with a PAPE strategy may depend simultaneously on the target task, caffeine dose, conditioning stimulus, expected competition time, and the athlete’s individual sensitivity of sleep to caffeine.

The present study has several methodological strengths. The randomized, double-blind, placebo-controlled crossover design allowed each athlete to serve as his own control, thereby reducing the influence of between-participant differences in baseline neuromuscular capacity on the CAF–PLA comparison; this is particularly relevant for post-conditioning performance responses, which are themselves characterized by substantial inter-individual variability. In addition, the present study did not prescribe a uniform DJ height to all participants, but instead used multi-height testing to identify the drop height associated with each athlete’s peak RSI and maintained the same individualized prescription under both CAF and PLA. The statistical analysis further retained all valid post-randomization observations and controlled for McKay training/performance tier, Period, and Sequence, while accounting for repeated-measures dependence through participant- and session-level random effects and a CAR(1) residual structure. The overall inference remained consistent across Pre-CA-adjusted, DXA-adjusted, and complete-case analyses, and leave-one-participant-out analyses likewise did not indicate that the principal inference was driven by any single individual. Together, these features strengthen the consistency of the main conclusion across different analytical specifications. Nevertheless, an important methodological consideration inherent to crossover designs is the possibility of a carryover effect, whereby the effect of a treatment administered in the first period persists into the subsequent period and may influence the within-participant treatment comparison [47,48]. Methodological guidance for randomized crossover trials therefore emphasizes the use of an adequately justified washout interval to minimize such residual influences, while recognizing that the absence of statistically significant period- or sequence-related effects should not, by itself, be interpreted as definitive proof that carryover is completely absent [47,48]. In the present study, a 72-h washout interval was selected because it substantially exceeds the average elimination half-life of caffeine in healthy adults (approximately 4–6 h), while also allowing recovery from the preceding individualized DJ conditioning activity and repeated maximal CMJ assessments [9]. Consistent with this design rationale, neither Period nor Sequence showed a statistically clear effect in the Pre-CA analysis or in the primary post-conditioning mixed-effects model, and the period-specific Pre-CA CMJ values did not show a statistically clear CAF–PLA difference. Collectively, these findings provide no evident signal of a material carryover effect under the present experimental conditions. Nevertheless, caffeine pharmacokinetics show considerable inter-individual variability, and residual treatment effects cannot be excluded with absolute certainty solely on the basis of the washout interval and statistical adjustment. Accordingly, the present findings should be interpreted as showing no detectable evidence of a meaningful carryover effect under the 72-h washout protocol, rather than as demonstrating the complete absence of carryover.

Several limitations should also be acknowledged. First, both conditions included the same RSI-individualized DJ conditioning activity and no no-conditioning control was included; therefore, the present design identifies the additional effect of CAF relative to PLA within a shared conditioning context, but cannot independently separate the effect of CAF, the effect of DJ conditioning, and their true interaction. Second, Pre-CA was measured after rather than before supplement ingestion and therefore represents a “post-supplement, pre-conditioning” reference rather than a true pre-supplementation baseline; consequently, the present study cannot reconstruct the complete acute effect of caffeine from before ingestion. Third, the sample size was determined for the primary Condition × Time hypothesis rather than for the three-way interaction, and the precision of the habitual-caffeine-exposure moderation analyses was therefore limited. Caffeine exposure was estimated primarily from self-reported intake over the previous 4 weeks, and no additional biochemical verification was undertaken; the uniform 24-h caffeine-abstinence procedure may also have induced some degree of withdrawal-related response in regular consumers, although such symptoms were not systematically recorded. In addition, although coffee, tea, energy drinks, and caffeinated soft drinks all contributed to habitual caffeine intake, these products also differ in their non-caffeine bioactive constituents and accompanying nutrients. Accordingly, the exposure variable in the present study is more appropriately interpreted as an estimate of habitual caffeine intake, and may not fully capture the broader physiological exposure associated with different caffeine-containing products. Finally, the study included only Chinese Han male basketball players, primarily classified as McKay Tier 2–3, and caution is therefore required when generalizing these findings to female athletes, higher-performance-caliber athletes, individuals of other ancestry, or other sports.

However, the present study further defines the boundary of combining acute caffeine supplementation with an individualized conditioning strategy. Following RSI-individualized DJ conditioning, 3 mg·kg^−1^ caffeine did not produce a statistically clear additional change in the temporal CMJ response; this overall conclusion remained consistent across the primary, supportive, and multiple sensitivity analyses, while habitual caffeine exposure likewise did not show a clear global moderating effect. Considered together with the additional CAF benefit reported by Guerra et al. [37] and the absence of a clear additional CAF effect reported by Filip-Stachnik et al. [12], the current evidence more strongly supports the view that there is no fixed caffeine–conditioning synergy that applies universally across contexts; rather, their combined effect is more likely to be jointly determined by caffeine dose, conditioning-stimulus characteristics, performance task, recovery time, and athlete-specific characteristics. More importantly, the present study suggests that individualizing the conditioning height does not automatically individualize the recovery window. Future studies should therefore move beyond a simple “CAF vs. PLA” comparison by using factorial designs such as CAF + conditioning, PLA + conditioning, CAF + no-conditioning, and PLA + no-conditioning to directly disentangle the independent and interactive effects of the two strategies, while using larger samples to further investigate individualized matching among conditioning dose, caffeine exposure, and the post-conditioning recovery window.

## 5. Conclusions

In Chinese Han male basketball players, this study did not obtain clear evidence that acute ingestion of 3 mg·kg^−1^ caffeine provided an additional improvement in CMJ performance following the specific 3 × 5 RSI-individualized drop-jump conditioning protocol. Although CMJ performance varied across post-conditioning recovery time, neither the primary Condition × Time interaction nor the Holm-adjusted time-specific contrasts demonstrated a consistent CAF advantage, and this inference remained stable across supportive and sensitivity analyses. Habitual caffeine exposure, whether modeled continuously or categorically, did not show a significant global moderating effect; therefore, isolated time-specific signals should be regarded as exploratory and hypothesis-generating rather than evidence of a consistent subgroup effect. The timing of maximum observed ΔCMJ also remained heterogeneous despite individualization of drop height, indicating that individualizing the conditioning stimulus does not necessarily individualize the recovery window. Future larger factorial studies incorporating no-conditioning conditions are needed to disentangle the independent and interactive effects of caffeine and conditioning and to refine individualized stimulus–recovery strategies.

## Figures and Tables

**Table 1:** Participant characteristics in the randomized available-data and complete-case cohorts by reported caffeine exposure and intervention sequence. Panel (**A**) Randomized available-data cohort; Panel (**B**) Complete-case cohort.

Variable	Total	Reported Caffeine Exposure				Intervention Sequence			
		No Reported Exposure	Reported Exposure	*p*	ES	CAF→PLA	PLA→CAF	*p*	ES
(**A**)									
	**(*n* = 30)**	**(*n* = 17)**	**(*n* = 13)**			**(*n* = 15)**	**(*n* = 15)**		
Age, y	22.00 [21.00, 27.75]	22.00 [20.00, 25.00]	23.00 [21.00, 29.00]	0.263 †	0.244	22.00 [20.50, 23.00]	26.00 [21.00, 29.00]	0.132 †	0.324
Height, cm	181.70 ± 7.36	182.71 ± 8.36	180.38 ± 5.88	0.402 ‡	−0.314	181.53 ± 7.15	181.87 ± 7.82	0.904 ‡	0.044
Body mass, kg	77.54 ± 9.64	76.87 ± 10.60	78.42 ± 8.57	0.670 ‡	0.159	74.87 ± 9.33	80.22 ± 9.49	0.131 ‡	0.569
BMI, kg·m^−2^	23.69 [22.69, 24.90]	23.66 [21.50, 24.90]	23.89 [22.99, 24.78]	0.530 †	0.140	23.45 [22.67, 24.53]	24.49 [22.92, 26.38]	0.165 †	0.302
McKay Tier 2, *n* (%)	13 (43.3)	4 (23.5)	9 (69.2)	0.025 §	0.457	4 (26.7)	9 (60.0)	0.139 §	0.336
McKay Tier 3, *n* (%)	17 (56.7)	13 (76.5)	4 (30.8)			11 (73.3)	6 (40.0)		
Habitual caffeine intake, mg·week^−1^	0.00 [0.00, 220.38]	0.00 [0.00, 0.00]	420.00 [100.00, 504.00]	<0.001 †	1.000	0.00 [0.00, 16.50]	220.00 [0.00, 472.50]	0.018 †	0.462
Habitual caffeine intake, mg·day^−1^	0.00 [0.00, 31.48]	0.00 [0.00, 0.00]	60.00 [14.29, 72.00]	<0.001 †	1.000	0.00 [0.00, 2.36]	31.43 [0.00, 67.50]	0.018 †	0.462
Habitual caffeine intake, mg·kg^−1^·day^−1^	0.00 [0.00, 0.42]	0.00 [0.00, 0.00]	0.80 [0.19, 0.91]	<0.001 †	1.000	0.00 [0.00, 0.03]	0.41 [0.00, 0.83]	0.031 †	0.422
DXA body fat, %	16.15 [14.62, 18.75]	16.20 [14.70, 17.70]	16.00 [14.60, 19.10]	0.983 †	−0.009	16.60 [15.35, 18.70]	15.50 [14.25, 18.35]	0.340 †	−0.209
DXA-derived lean mass, kg	62.14 ± 6.65	62.01 ± 7.02	62.31 ± 6.42	0.903 ‡	0.045	60.85 ± 6.02	63.44 ± 7.19	0.294 ‡	0.391
DXA BMD, g·cm^−2^	1.34 ± 0.13	1.33 ± 0.11	1.35 ± 0.15	0.687 ‡	0.150	1.33 ± 0.11	1.35 ± 0.15	0.672 ‡	0.156
(**B**)									
	**(*n* = 27)**	**(*n* = 17)**	**(*n* = 10)**			**(*n* = 14)**	**(*n* = 13)**		
Age, y	22.00 [20.50, 28.00]	22.00 [20.00, 25.00]	22.50 [21.00, 29.00]	0.347 †	0.224	22.00 [20.25, 23.50]	23.00 [21.00, 29.00]	0.291 †	0.242
Height, cm	181.15 ± 7.40	182.71 ± 8.36	178.50 ± 4.65	0.158 ‡	−0.581	180.93 ± 7.01	181.38 ± 8.08	0.877 ‡	0.060
Body mass, kg	76.81 ± 9.52	76.87 ± 10.60	76.70 ± 7.89	0.965 ‡	−0.018	74.29 ± 9.40	79.52 ± 9.24	0.157 ‡	0.562
BMI, kg·m^−2^	23.72 [22.67, 24.90]	23.66 [21.50, 24.90]	24.19 [22.88, 24.73]	0.564 †	0.141	23.45 [22.66, 24.71]	24.49 [22.69, 26.59]	0.244 †	0.269
McKay Tier 2, *n* (%)	11 (40.7)	4 (23.5)	7 (70.0)	0.040 §	0.457	4 (28.6)	7 (53.8)	0.252 §	0.257
McKay Tier 3, *n* (%)	16 (59.3)	13 (76.5)	3 (30.0)			10 (71.4)	6 (46.2)		
Habitual caffeine intake, mg·week^−1^	0.00 [0.00, 160.00]	0.00 [0.00, 0.00]	320.25 [130.00, 483.00]	<0.001 †	1.000	0.00 [0.00, 0.00]	100.00 [0.00, 420.00]	0.044 †	0.401
Habitual caffeine intake, mg·day^−1^	0.00 [0.00, 22.86]	0.00 [0.00, 0.00]	45.75 [18.57, 69.00]	<0.001 †	1.000	0.00 [0.00, 0.00]	14.29 [0.00, 60.00]	0.044 †	0.401
Habitual caffeine intake, mg·kg^−1^·day^−1^	0.00 [0.00, 0.30]	0.00 [0.00, 0.00]	0.61 [0.24, 0.91]	<0.001 †	1.000	0.00 [0.00, 0.00]	0.16 [0.00, 0.80]	0.061 †	0.374
DXA body fat, %	16.40 [15.10, 19.15]	16.20 [14.70, 17.70]	17.10 [15.62, 19.18]	0.514 †	0.159	17.75 ± 4.74	17.11 ± 4.63	0.725 ‡	−0.137
DXA-derived lean mass, kg	61.42 ± 6.51	62.01 ± 7.02	60.41 ± 5.76	0.548 ‡	−0.243	60.40 ± 5.99	62.51 ± 7.11	0.412 ‡	0.321
DXA BMD, g·cm^−2^	1.33 ± 0.12	1.33 ± 0.11	1.32 ± 0.14	0.898 ‡	−0.052	1.33 ± 0.11	1.32 ± 0.14	0.841 ‡	−0.078

Panel (**A**), randomized available-data cohort (*n* = 30); Panel (**B**), complete-case cohort (*n* = 27). Data are mean ± SD, median [Q1, Q3], or *n* (%). † Mann–Whitney U test (rank-biserial r); ‡ independent-samples *t* test (Cohen’s d); § Fisher’s exact test (Cramér’s V). BMI, body mass index; DXA, dual-energy X-ray absorptiometry; BMD, bone mineral density; CAF, caffeine; PLA, placebo; ES, effect size.

**Table 2:** Pre-CA CMJ analysis after supplement ingestion and before conditioning. Panel (**A**) Adjusted means and CAF–PLA contrast; Panel (**B**) Omnibus model tests.

(**A**)				
**Condition**	**Estimate (95% CI), cm**		**SE**	***df***
PLA	45.65 (43.61 to 47.70)		0.99	25
CAF	46.10 (44.04 to 48.16)		1.00	25
CAF–PLA	0.45 (−0.58 to 1.48)		0.50	25
(**B**)				
**Effect**	**Num *df***	**Den *df***	**F**	***p***
Condition	1	25	0.798	0.380
McKay tier	1	27	1.072	0.310
Period	1	25	0.180	0.675
Sequence	1	27	0.178	0.676

Pre-CA was measured after CAF/PLA ingestion and before conditioning and was analyzed separately from the primary model. CAF, caffeine; PLA, placebo; CMJ, countermovement jump; CI, confidence interval; SE, standard error; *df*, degrees of freedom.

**Table 3:** Primary mixed-effects analysis of post-conditioning CMJ performance. Panel (**A**) Omnibus fixed-effect tests; Panel (**B**) Time-specific adjusted means and CAF–PLA contrasts.

(**A**)					
**Effect**	**Num *df***	**Den *df***		**F**	***p***
Condition	1	25		3.333	0.080
Time	4	220		7.708	<0.001
McKay tier	1	27		1.216	0.280
Period	1	25		0.017	0.897
Sequence	1	27		0.191	0.665
Condition × Time	4	220		1.216	0.305
(**B**)					
**Time**	**PLA Adjusted Mean (95% CI)**	**CAF Adjusted Mean (95% CI)**	**CAF–PLA (95% CI), cm**	**Std. Effect**	**Holm *p***
15 s	45.50 (43.36 to 47.64)	46.38 (44.23 to 48.53)	0.89 (−0.11 to 1.88)	0.61	0.399
3 min	46.19 (44.05 to 48.34)	46.58 (44.43 to 48.73)	0.39 (−0.61 to 1.38)	0.26	0.868
6 min	45.38 (43.24 to 47.52)	45.58 (43.43 to 47.73)	0.21 (−0.79 to 1.21)	0.14	0.868
9 min	44.64 (42.50 to 46.78)	45.49 (43.34 to 47.64)	0.84 (−0.15 to 1.84)	0.58	0.399
12 min	44.43 (42.29 to 46.57)	45.14 (42.99 to 47.29)	0.71 (−0.29 to 1.71)	0.49	0.464

Estimates are from the primary available-data mixed-effects model. Standardized effects equal the CAF–PLA contrast divided by the model residual SD; Holm adjustment was applied across the five time-specific contrasts. CAF, caffeine; PLA, placebo; CMJ, countermovement jump; CI, confidence interval; *df*, degrees of freedom.

**Table 4:** Exploratory caffeine-exposure moderation analyses. Panel (**A**) Global three-way interaction tests; Panel (**B**) Continuous-exposure selected contrasts; Panel (**C**) Binary reported-exposure contrasts.

(**A**)					
**Exposure Representation**	**Global Interaction**	**Num *df***	**Den *df***	**F**	***p***
Continuous exposure	Condition × Time × exposure	4	212	1.672	0.158
Binary reported exposure	Condition × Time × group	4	212	1.872	0.116
(**B**)					
**Exposure Level**	**mg·kg^−1^·day^−1^**	**Time**	**CAF–PLA (95% CI), cm**	**Raw *p***	**Holm *p* (15)**
No reported exposure	0.00	15 s	1.13 (0.04 to 2.22)	0.043	0.559
No reported exposure	0.00	3 min	0.86 (−0.24 to 1.95)	0.119	0.831
No reported exposure	0.00	6 min	1.00 (−0.09 to 2.09)	0.071	0.749
No reported exposure	0.00	9 min	1.83 (0.74 to 2.92)	0.002	0.031
No reported exposure	0.00	12 min	1.61 (0.52 to 2.70)	0.006	0.078
Median non-zero exposure	0.80	15 s	0.34 (−1.14 to 1.82)	0.639	1.000
Median non-zero exposure	0.80	3 min	−0.54 (−2.02 to 0.94)	0.458	1.000
Median non-zero exposure	0.80	6 min	−1.37 (−2.85 to 0.11)	0.068	0.749
Median non-zero exposure	0.80	9 min	−1.12 (−2.61 to 0.36)	0.131	0.831
Median non-zero exposure	0.80	12 min	−1.12 (−2.60 to 0.37)	0.133	0.831
90th percentile exposure	0.91	15 s	0.26 (−1.36 to 1.89)	0.742	1.000
90th percentile exposure	0.91	3 min	−0.68 (−2.31 to 0.94)	0.395	1.000
90th percentile exposure	0.91	6 min	−1.61 (−3.24 to 0.01)	0.052	0.621
90th percentile exposure	0.91	9 min	−1.42 (−3.05 to 0.20)	0.083	0.750
90th percentile exposure	0.91	12 min	−1.39 (−3.02 to 0.23)	0.090	0.750
(**C**)					
**Group**		**Time**	**CAF–PLA (95% CI), cm**	**Raw *p***	**Holm *p* (10)**
No reported caffeine exposure		15 s	0.38 (−0.93 to 1.69)	0.553	1.000
No reported caffeine exposure		3 min	0.38 (−0.93 to 1.69)	0.559	1.000
No reported caffeine exposure		6 min	0.55 (−0.76 to 1.86)	0.397	1.000
No reported caffeine exposure		9 min	1.35 (0.04 to 2.66)	0.044	0.435
No reported caffeine exposure		12 min	1.16 (−0.15 to 2.47)	0.080	0.644
Reported caffeine exposure		15 s	1.62 (−0.06 to 3.29)	0.057	0.517
Reported caffeine exposure		3 min	0.38 (−1.29 to 2.06)	0.639	1.000
Reported caffeine exposure		6 min	−0.31 (−1.98 to 1.36)	0.704	1.000
Reported caffeine exposure		9 min	0.07 (−1.60 to 1.74)	0.932	1.000
Reported caffeine exposure		12 min	0.02 (−1.65 to 1.69)	0.981	1.000

Continuous exposure was log(1 + x)-transformed and z-standardized. Holm adjustment was applied across 15 contrasts in Panel (**B**) and 10 contrasts in Panel (**C**). Global three-way interactions take precedence over time-specific comparisons. CAF, caffeine; PLA, placebo; CI, confidence interval; *df*, degrees of freedom.

**Table 5:** Sensitivity analyses of the primary CMJ contrasts.

Model	Time	CAF–PLA (95% CI), cm	Std. Effect	Holm *p*	Global C × T *p*
Pre-CA adjusted	15 s	0.38 (−0.59 to 1.35)	0.26	1.000	0.304
Pre-CA adjusted	3 min	−0.12 (−1.09 to 0.85)	−0.08	1.000	0.304
Pre-CA adjusted	6 min	−0.30 (−1.27 to 0.67)	−0.21	1.000	0.304
Pre-CA adjusted	9 min	0.34 (−0.64 to 1.31)	0.23	1.000	0.304
Pre-CA adjusted	12 min	0.20 (−0.77 to 1.18)	0.14	1.000	0.304
DXA adjusted	15 s	0.89 (−0.11 to 1.89)	0.61	0.388	0.305
DXA adjusted	3 min	0.39 (−0.61 to 1.39)	0.27	0.852	0.305
DXA adjusted	6 min	0.21 (−0.79 to 1.21)	0.15	0.852	0.305
DXA adjusted	9 min	0.85 (−0.15 to 1.85)	0.58	0.388	0.305
DXA adjusted	12 min	0.72 (−0.28 to 1.72)	0.49	0.452	0.305
Complete case	15 s	0.78 (−0.22 to 1.79)	0.54	0.516	0.361
Complete case	3 min	0.42 (−0.58 to 1.43)	0.29	0.792	0.361
Complete case	6 min	0.15 (−0.85 to 1.16)	0.11	0.792	0.361
Complete case	9 min	0.82 (−0.18 to 1.83)	0.57	0.516	0.361
Complete case	12 min	0.77 (−0.24 to 1.77)	0.53	0.516	0.361

Pre-CA adjusted, conditional on post-supplement Pre-CA CMJ; DXA adjusted, additionally controlling for z-standardized lean mass and body-fat percentage; complete case, participants completing both periods (*n* = 27). C × T, Condition × Time; CAF, caffeine; PLA, placebo; CI, confidence interval.

**Table 6:** Covariance-structure comparison for the primary CMJ model.

Model	AIC	BIC	logLik
Nested random intercepts + CAR(1)	1093.95	1155.25	−529.97
Nested random intercepts only	1129.42	1187.11	−548.71

Both models used the same fixed effects and nested participant/session random intercepts; the first additionally included continuous-time AR(1) residual correlation. AIC, Akaike information criterion; BIC, Bayesian information criterion; logLik, log-likelihood; AR(1), first-order autoregressive.

**Table 7:** Supportive ΔCMJ analysis. Panel (**A**) Omnibus fixed-effect tests; Panel (**B**) Adjusted ΔCMJ and time-specific CAF–PLA contrasts.

(**A**)					
**Effect**	**Num *df***	**Den *df***		**F**	***p***
Condition	1	25		0.574	0.456
Time	4	220		7.682	<0.001
McKay tier	1	27		0.485	0.492
Period	1	25		1.060	0.313
Sequence	1	27		0.074	0.788
Condition × Time	4	220		1.219	0.304
(**B**)					
**Time**	**PLA ΔCMJ (95% CI)**	**CAF ΔCMJ (95% CI)**	**CAF–PLA (95% CI), cm**	**Std. Effect**	**Holm *p***
15 s	−0.18 (−0.89 to 0.52)	0.17 (−0.55 to 0.90)	0.36 (−0.62 to 1.34)	0.24	1.000
3 min	0.51 (−0.20 to 1.22)	0.37 (−0.35 to 1.10)	−0.14 (−1.12 to 0.84)	−0.10	1.000
6 min	−0.31 (−1.01 to 0.40)	−0.63 (−1.35 to 0.10)	−0.32 (−1.30 to 0.66)	−0.22	1.000
9 min	−1.04 (−1.75 to −0.33)	−0.72 (−1.45 to 0.00)	0.32 (−0.66 to 1.30)	0.22	1.000
12 min	−1.25 (−1.96 to −0.54)	−1.06 (−1.79 to −0.34)	0.19 (−0.79 to 1.16)	0.13	1.000

ΔCMJ was calculated relative to post-supplement Pre-CA and analyzed as a supportive outcome. CAF, caffeine; PLA, placebo; CMJ, countermovement jump; CI, confidence interval; *df*, degrees of freedom.

**Table 8:** Exploratory derived CMJ outcomes. Panel (**A**) Paired derived-outcome comparisons; Panel (**B**) Distribution of time to maximum observed ΔCMJ.

(**A**)								
**Outcome**	***n***	**PLA Mean**	**CAF Mean**	**CAF–PLA Summary**	**Test**	**Raw *p***	**Holm *p***	**Effect Size**
Maximum observed ΔCMJ	27	0.78	1.06	Median paired difference = −0.20	Wilcoxon signed-rank test	0.962	1.000	
ΔCMJ AUC (0–12 min)	27	−6.17	−4.34	1.82 (−7.95 to 11.60)	Paired *t* test	0.704	1.000	Cohen’s dz = 0.07
Time to maximum observed ΔCMJ	27	2.98	3.77	Median paired difference = 0.00 min	Paired Wilcoxon signed-rank test	0.442	1.000	
(**B**)								
**Condition**				**Time**	***n***	**%**		
PLA				15 s	10	34.5		
PLA				3 min	12	41.4		
PLA				6 min	4	13.8		
PLA				9 min	2	6.9		
PLA				12 min	1	3.4		
CAF				15 s	12	42.9		
CAF				3 min	8	28.6		
CAF				6 min	1	3.6		
CAF				9 min	5	17.9		
CAF				12 min	2	7.1		

Maximum observed ΔCMJ is the largest value among the five prespecified post-conditioning time points; time to maximum observed ΔCMJ is therefore discrete. Panel (**A**) includes paired complete cases (*n* = 27); Panel (**B**) includes all valid condition-specific sessions. CAF, caffeine; PLA, placebo; CMJ, countermovement jump; AUC, area under the curve.

**Table 9:** Secondary HR and RPE analyses. Panel (**A**) Omnibus fixed-effect tests; Panel (**B**) Adjusted means and time-specific CAF–PLA contrasts.

(**A**)						
**Outcome**	**Effect**	**Num *df***		**Den *df***	**F**	***p***
HR	Baseline HR	1		24	9.566	0.005
HR	Condition	1		24	1.779	0.195
HR	Time	4		220	83.202	<0.001
HR	McKay tier	1		27	0.478	0.495
HR	Period	1		24	0.025	0.877
HR	Sequence	1		27	0.056	0.814
HR	Condition × Time	4		220	0.476	0.754
RPE	Baseline RPE	1		24	63.828	<0.001
RPE	Condition	1		24	6.508	0.018
RPE	Time	4		220	25.570	<0.001
RPE	McKay tier	1		27	9.187	0.005
RPE	Period	1		24	0.109	0.744
RPE	Sequence	1		27	2.216	0.148
RPE	Condition × Time	4		220	1.349	0.253
(**B**)						
**Outcome**	**Time**	**PLA Adjusted Mean** **(95% CI)**	**CAF Adjusted Mean** **(95% CI)**	**CAF–PLA (95% CI)**	**Std. Effect**	**Holm *p***
HR	15 s	115.11 (110.51 to 119.70)	112.45 (107.81 to 117.10)	−2.65 (−6.76 to 1.45)	−0.37	0.778
HR	3 min	100.07 (95.48 to 104.66)	97.35 (92.70 to 101.99)	−2.73 (−6.83 to 1.38)	−0.38	0.778
HR	6 min	93.73 (89.14 to 98.32)	90.81 (86.17 to 95.45)	−2.92 (−7.02 to 1.19)	−0.40	0.778
HR	9 min	91.35 (86.76 to 95.94)	90.13 (85.49 to 94.77)	−1.22 (−5.32 to 2.89)	−0.17	1.000
HR	12 min	89.97 (85.38 to 94.56)	89.63 (84.99 to 94.27)	−0.34 (−4.44 to 3.77)	−0.05	1.000
RPE	15 s	4.94 (4.51 to 5.37)	4.33 (3.89 to 4.77)	−0.60 (−1.09 to −0.12)	−0.68	0.088
RPE	3 min	3.80 (3.37 to 4.23)	3.50 (3.06 to 3.93)	−0.31 (−0.79 to 0.18)	−0.35	0.837
RPE	6 min	3.49 (3.06 to 3.92)	3.32 (2.88 to 3.76)	−0.17 (−0.66 to 0.32)	−0.20	1.000
RPE	9 min	3.39 (2.96 to 3.82)	3.39 (2.95 to 3.83)	0.00 (−0.49 to 0.49)	0.00	1.000
RPE	12 min	3.46 (3.03 to 3.89)	3.39 (2.95 to 3.83)	−0.07 (−0.56 to 0.42)	−0.08	1.000

HR and RPE were secondary outcomes; models adjusted for the corresponding post-supplement baseline and used the primary repeated-measures structure. Holm adjustment was applied across the five time-specific contrasts for each outcome. HR, heart rate; RPE, rating of perceived exertion; CAF, caffeine; PLA, placebo; CI, confidence interval; *df*, degrees of freedom.

## Data Availability

The datasets generated and/or analyzed during the current study are not publicly available due to ethical restrictions and participant privacy protection. However, anonymized data may be available from the corresponding author upon reasonable request and subject to approval by the institutional ethics committee.

## Abbreviations

The following abbreviations are used in this manuscript:

1RM	One-repetition maximum
ADORA2A	Adenosine A2A receptor
AI	Artificial intelligence
AIC	Akaike information criterion
AR(1)	First-order autoregressive
ATP	Adenosine triphosphate
AUC	Area under the curve
BIC	Bayesian information criterion
BMD	Bone mineral density
BMI	Body mass index
CA	Conditioning activity
CAF	Caffeine
CAR(1)	Continuous-time first-order autoregressive
CI	Confidence interval
CMJ	Countermovement jump
CONSORT	Consolidated Standards of Reporting Trials
CR10	Category-Ratio 10 scale
CV	Coefficient of variation
CYP1A2	Cytochrome P450 family 1 subfamily A member 2
*df*	Degrees of freedom
DJ	Drop jump
DXA	Dual-energy X-ray absorptiometry
ES	Effect size
HR	Heart rate
ICC	Intraclass correlation coefficient
ICC(A,1)	Intraclass correlation coefficient, absolute-agreement, single-measure
IQR	Interquartile range
logLik	Log-likelihood
ODH	Optimal drop height
PAPE	Post-activation performance enhancement
PCr	Phosphocreatine
PLA	Placebo
Pre-CA	Pre-conditioning activity reference measurement
Q1	First quartile
Q3	Third quartile
RPE	Rating of perceived exertion
RSI	Reactive strength index
SD	Standard deviation
SE	Standard error
SMD	Standardized mean difference
SWC	Smallest worthwhile change
TE	Typical error
ΔCMJ	Change in countermovement jump relative to Pre-CA

## Appendix A

**Table A1:** Standardized AI-Assisted Protocol for Habitual Caffeine Exposure Coding and Participant-Level Input Template.

Section	Component	Standardized Instruction/Operational Rule
Overview	Purpose	Presents the fixed standardized instruction set used for AI-assisted extraction, coding, calculation, and quality control of habitual caffeine exposure data, followed by the participant-level input template applied consistently for each individual analysis. All AI-generated outputs require researcher verification before inclusion in the final research dataset.
Part A	Role definition and task scope	Role: “Standardized Habitual Caffeine Exposure Coding Assistant” for research data processing. Task: Use the Habitual Caffeine Exposure Questionnaire for the Previous 4 Weeks to standardize, extract, code, and calculate information from free-text responses, product information, serving amounts, and consumption frequencies.
Part A	Responsibilities	Structure and extract questionnaire information. Standardize product names and consumption frequencies. Convert caffeine doses only from caffeine-content information provided or verified by the research team. Calculate average habitual caffeine exposure over the previous 4 weeks. Flag all information requiring manual review.
Part A	Critical restriction	The AI is strictly prohibited from using its own knowledge, general assumptions, or unverified memory to guess the caffeine content of any product.
I. Input Information	Participant information and screening	Required participant fields: Participant ID; DXA body mass (kg). Overall screening question: Did the participant consume any identifiable caffeine-containing food or beverage during the previous 4 weeks? Response options: No/Yes.
I. Input Information	Item-level intake records	Each product record may contain: product name, brand, or category; package size or usual serving size; number of servings consumed per occasion; average consumption frequency; frequency unit (/day,/week, or/4 weeks); whether consumption frequency varied over the 4-week period; notes.
I. Input Information	Researcher-verified caffeine reference information	May include: standardized product name; product size; caffeine content; caffeine-content basis (e.g., mg/bottle, mg/can, mg/cup, mg/serving, mg/100 mL); information source (product label, manufacturer information, or standardized reference value predefined by the research team).
II. General Processing Principles	Assessment and information-use rules	Fix the assessment window as the previous 4 consecutive weeks. Use images only as visual memory cues; they are not an exhaustive product list. Do not infer exact brand, product size, or caffeine content solely from questionnaire images. Prioritize participant-reported specific brand, product name, and product size when matching products. If a product cannot be identified reliably, retain the original description and set manual_review_required = YES. If verified caffeine-content information is unavailable, do not estimate independently; set caffeine_value_status = “unresolved” and needs_manual_lookup = YES. Retain all original information; do not overwrite or delete it during standardized coding. Flag ambiguity, inconsistency, or uncertainty for manual review rather than resolving it through subjective inference.
III. Item-Level Product Standardization	Required output fields	For each intake record, generate: item_id; raw_product_description; standardized_product_name; product_category; package_or_usual_serving; servings_per_occasion; average_frequency_value; frequency_unit; varied_over_4_weeks; caffeine_value; caffeine_value_unit; caffeine_value_basis; caffeine_source; manual_review_required.
III. Item-Level Product Standardization	Controlled categories and allowed values	Product category: Coffee; Tea/Tea-based beverages; Energy drinks; Caffeinated soft drinks; Chocolate/Cocoa products; Other caffeine-containing products; Unresolved. frequency_unit allowed values: per_day; per_week; per_4_weeks. varied_over_4_weeks allowed values: YES; NO; UNKNOWN. raw_product_description must retain the participant’s original response exactly as recorded.
IV. Frequency Standardization Rules	Conversion to average weekly frequency	Times per day: weekly_frequency = average_frequency_value × 7. Times per week: weekly_frequency = average_frequency_value. Times per 4 weeks: weekly_frequency = average_frequency_value ÷ 4. Examples: 2 times/day → 14 times/week; 3 times/week → 3 times/week; 5 times/4 weeks → 1.25 times/week. If frequency varied over the previous 4 weeks, use the reported average frequency across the 4-week period and retain varied_over_4_weeks = YES.
V. Caffeine Content Matching Principles	Priority order	Priority 1: Product-label information exactly matching the product consumed. Priority 2: Official information published by the manufacturer. Priority 3: A product database previously verified and fixed by the research team. Priority 4: For foods or beverages without identifiable product-specific information, standardized reference values predefined by the research team.
V. Caffeine Content Matching Principles	Prohibited actions and unresolved values	Do not generate caffeine values from the AI model’s internal knowledge; guess caffeine content based on brand familiarity; extrapolate caffeine content from similar products; or assume package volume when product size is missing. If caffeine content cannot be determined reliably, output: caffeine_value = NA; needs_manual_lookup = YES; manual_review_required = YES.
VI. Item-Level Caffeine Intake Calculation	Per-occasion and weekly calculations	caffeine_mg_per_occasion = caffeine_mg_per_serving × servings_per_occasion. item_caffeine_mg_week = caffeine_mg_per_occasion × weekly_frequency. If caffeine content is reported as mg/100 mL, convert according to the actual consumed volume only when both product volume and actual amount consumed are clearly available. Example: 32 mg/100 mL × 250 mL ÷ 100 = 80 mg per occasion.
VII. Total Habitual Caffeine Exposure	Participant-level calculations	Total caffeine intake (mg·week^−1^) = sum of item_caffeine_mg_week across all valid products. Mean daily caffeine intake (mg·day^−1^) = Total caffeine intake (mg·week^−1^) ÷ 7. Body-mass-adjusted caffeine intake (mg·kg^−1^·day^−1^) = Mean daily caffeine intake (mg·day^−1^) ÷ DXA body mass (kg).
VIII. Binary Habitual Caffeine Exposure Variable	Operational definition	If Total caffeine intake = 0 mg·week^−1^: binary_exposure_status = 0; exposure_group = “No habitual caffeine exposure”. If Total caffeine intake > 0 mg·week^−1^: binary_exposure_status = 1; exposure_group = “Habitual caffeine exposure”. If overall screening conflicts with item-level records, do not classify automatically; set manual_review_required = YES and inconsistency_flag = YES.
IX. Continuous Exposure Variable	Transformation and standardization	Primary continuous variable: habitual_caffeine_mg_kg_day (mg·kg^−1^·day^−1^). After all participants are coded: log_transformed_exposure = ln(1 + habitual_caffeine_mg_kg_day). Then standardize across the full cohort: z_exposure = [log_transformed_exposure − cohort mean] ÷ cohort standard deviation. Do not calculate the final z-score during single-participant processing; standardize only after full-sample processing.
X. Missing, Ambiguous, or Abnormal Data	Mandatory manual-review triggers	Manual review is required when: product name cannot be identified; brand or product size is unclear; serving amount is missing; consumption frequency is missing; frequency unit is unclear; caffeine-content source is unclear; product information does not match the reference database; overall screening and item-level records are inconsistent; calculated results appear implausible or abnormal; or completion would require inference or assumption. Do not impute unknown information solely to produce a complete result.
XI. Final Output Format	A. Item-Level Standardized Coding	For each item, report: Item ID; Raw product description; Standardized product name; Product category; Package/usual serving; Servings per occasion; Average frequency; Frequency unit; Weekly frequency; Caffeine value; Caffeine value basis; Caffeine source; Caffeine per occasion (mg); Estimated caffeine intake (mg·week^−1^); Needs manual lookup; Manual review required; Notes.
XI. Final Output Format	B. Participant-Level Exposure Summary	Report: Participant ID; DXA body mass (kg); Total caffeine intake (mg·week^−1^); Mean daily caffeine intake (mg·day^−1^); Body-mass-adjusted caffeine intake (mg·kg^−1^·day^−1^); Binary exposure status (0/1); Exposure group; log [1 + mg·kg^−1^·day^−1^]; Manual review required (YES/NO).
XI. Final Output Format	C. Quality-Control Report and final status	List separately: unidentified products; missing information; items with unresolved caffeine content; internal inconsistencies within the questionnaire; information requiring manual review because the AI could not determine it reliably. Final status options: Ready for researcher verification; Requires additional product lookup; Requires questionnaire clarification.
XII. Final Constraints	Researcher verification requirement	The AI output is an AI-assisted standardized coding result, not the final research dataset. All product identification, caffeine-content sources, frequency conversions, and final habitual caffeine exposure estimates must be manually checked by the research team against the original questionnaire before inclusion in the final study dataset. No AI-generated inference that has not undergone manual verification may be treated as final research data.
Part B. Fixed Input Template	Use instruction	Append the fixed input template after the standardized instructions for each participant-level analysis. Replace placeholders with participant-specific questionnaire data and researcher-verified caffeine reference information.
Part B. Fixed Input Template	Participant information and overall screening	Input Header: Participant Caffeine Exposure Data Processing. Fields: Participant ID; DXA body mass (kg). Screening: Did the participant report any caffeine intake during the previous 4 weeks? Response options: No/Yes.
Part B. Fixed Input Template	Questionnaire product records	Paste the complete original questionnaire records for the participant without altering the source responses.
Part B. Fixed Input Template	Verified caffeine reference data	For corresponding products, provide researcher-verified: Product name; Brand; Package size; Caffeine content; Caffeine-content basis (e.g., mg/serving, mg/100 mL); Information source (e.g., product label, manufacturer information, predefined research database).
Part B. Fixed Input Template	Processing instruction	Strictly follow the standardized coding instructions to complete: (1) structured extraction of questionnaire information; (2) product and consumption-frequency standardization; (3) caffeine-dose conversion based only on verified caffeine reference information; (4) calculation of habitual caffeine exposure during the previous 4 weeks; (5) quality-control assessment and identification of information requiring manual review.
Part B. Fixed Input Template	Uncertain information and required final output	For information that cannot be reliably confirmed: do not infer or estimate; do not use unverified assumptions; mark uniformly as requiring manual review. Provide final output according to the predefined reporting format, including item-level standardized coding, participant-level caffeine exposure summary, quality-control report, and final processing status.

**Table A2:** Within-session repeatability of duplicate countermovement-jump trials.

Condition	Time Point	*n*	Trial 1, Mean ± SD (cm)	Trial 2, Mean ± SD (cm)	Typical Error (cm)	CV (%)	ICC(A,1)
PLA	Pre-warm-up	29	40.96 ± 5.81	41.92 ± 5.40	1.01	2.44	0.955
PLA	Post-warm-up	29	43.96 ± 5.93	44.66 ± 5.66	1.14	2.56	0.956
PLA	15 s	29	44.34 ± 5.69	44.32 ± 5.95	0.89	2.02	0.977
PLA	3 min	29	44.87 ± 6.39	44.99 ± 5.68	1.24	2.75	0.959
PLA	6 min	29	44.15 ± 5.95	44.16 ± 5.96	1.13	2.56	0.965
PLA	9 min	29	43.63 ± 5.89	43.36 ± 5.61	1.17	2.70	0.959
PLA	12 min	29	43.17 ± 5.52	43.06 ± 5.57	1.30	3.03	0.946
CAF	Pre-warm-up	28	41.04 ± 6.28	41.65 ± 5.70	1.96	4.75	0.892
CAF	Post-warm-up	28	44.81 ± 5.84	45.79 ± 5.91	0.89	1.96	0.965
CAF	15 s	28	45.23 ± 6.22	45.91 ± 6.12	1.00	2.20	0.969
CAF	3 min	28	46.04 ± 6.00	45.79 ± 6.29	0.79	1.73	0.983
CAF	6 min	28	45.08 ± 5.97	45.28 ± 6.09	0.91	2.02	0.977
CAF	9 min	28	44.51 ± 5.99	44.23 ± 6.20	1.59	3.59	0.933
CAF	12 min	28	44.57 ± 6.07	43.82 ± 5.88	1.18	2.68	0.955

TE = SD(Trial 2 − Trial 1)/√2. CV (%) = 100 × TE/grand mean of Trial 1 and Trial 2. ICC(A,1) denotes a two-way mixed-effects, absolute-agreement, single-measure intraclass correlation coefficient. PLA, placebo; CAF, caffeine. Available paired duplicate observations were *n* = 29 under PLA and *n* = 28 under CAF; reliability estimates were calculated from complete paired Trial 1–Trial 2 observations at each time point.
